# Supplementary material for: Development and validation of the CHIME simulation model to assess lifetime health outcomes of prediabetes and type 2 diabetes in Chinese populations: A modeling study
Source: PLoS Med. 2021 Jun 24;18(6):e1003692. doi: 10.1371/journal.pmed.1003692 (PMC8270422; doi:10.1371/journal.pmed.1003692)
Supplement: S1 Fig — Predictions using CHIME are presented if available. Points are displayed for deciles of predicted and observed event rates, with fewer centiles than deciles used if fewer than 5 events were observed per group to prevent unstable inferences. CHARLS, China Health and Retirement Longitudinal Study; CHIME, Chinese Hong Kong Integrated Modeling and Evaluation; CMS, Clinical Management System. (DOCX) [file pmed.1003692.s002.docx]

S1 Figure. Calibration plots in prediabetes population


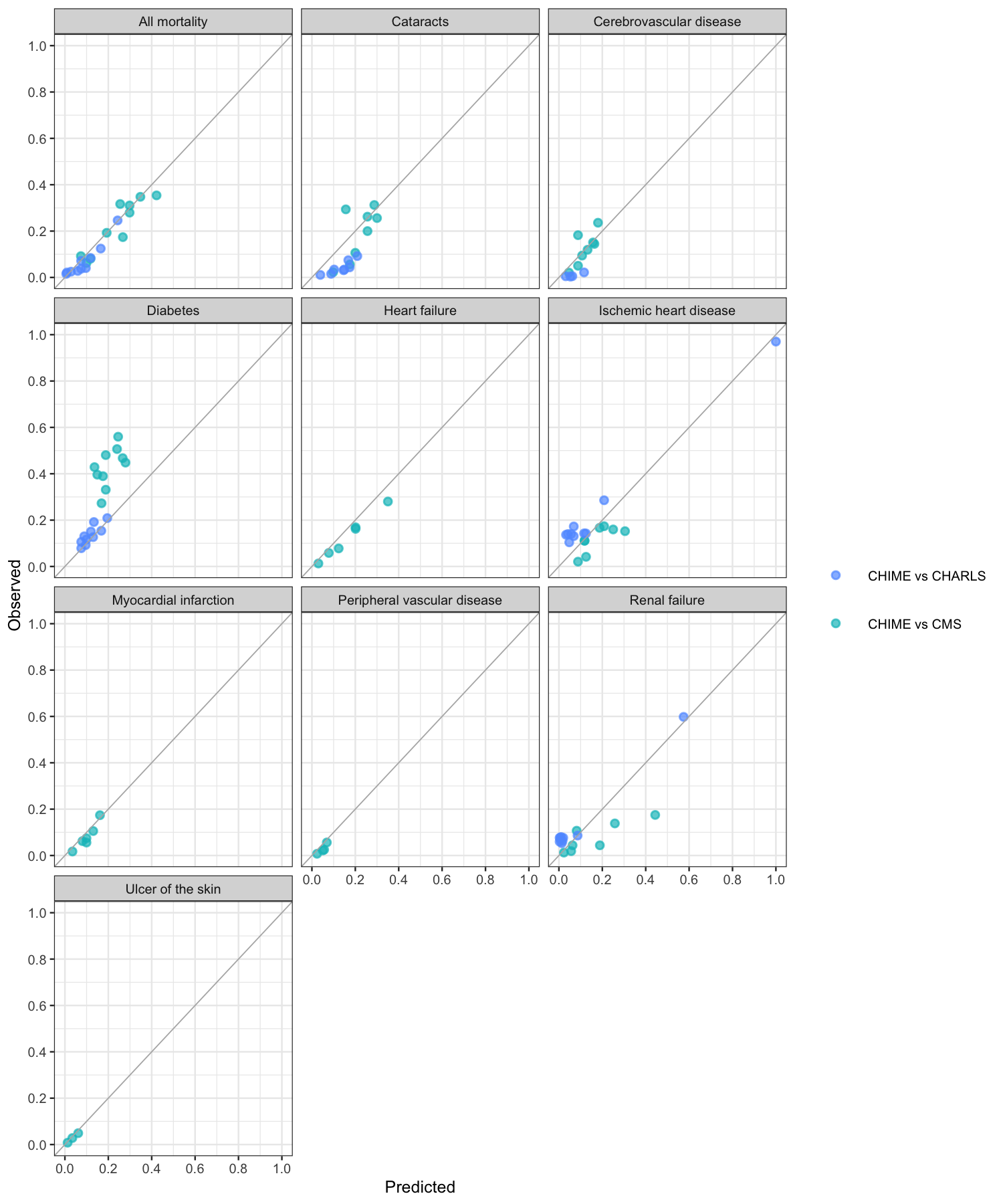


Predictions using CHIME are presented if available. Points are displayed for deciles of predicted and observed event rates, with fewer centiles than deciles used if fewer than 5 events were observed per group to prevent unstable inferences.

CHIME, Chinese Hong Kong Integrated Modelling and Evaluation.
