# Supplementary material for: Development and validation of the CHIME simulation model to assess lifetime health outcomes of prediabetes and type 2 diabetes in Chinese populations: A modeling study
Source: PLoS Med. 2021 Jun 24;18(6):e1003692. doi: 10.1371/journal.pmed.1003692 (PMC8270422; doi:10.1371/journal.pmed.1003692)
Supplement: S2 Fig — Predicted event rates from CHIME model against the observed events rates over time for CMS participants with prediabetes. CHIME, Chinese Hong Kong Integrated Modeling and Evaluation; CMS, Clinical Management System. (DOCX) [file pmed.1003692.s003.docx]

S2 Figure. Validation of CHIME against CMS cohort with prediabetes


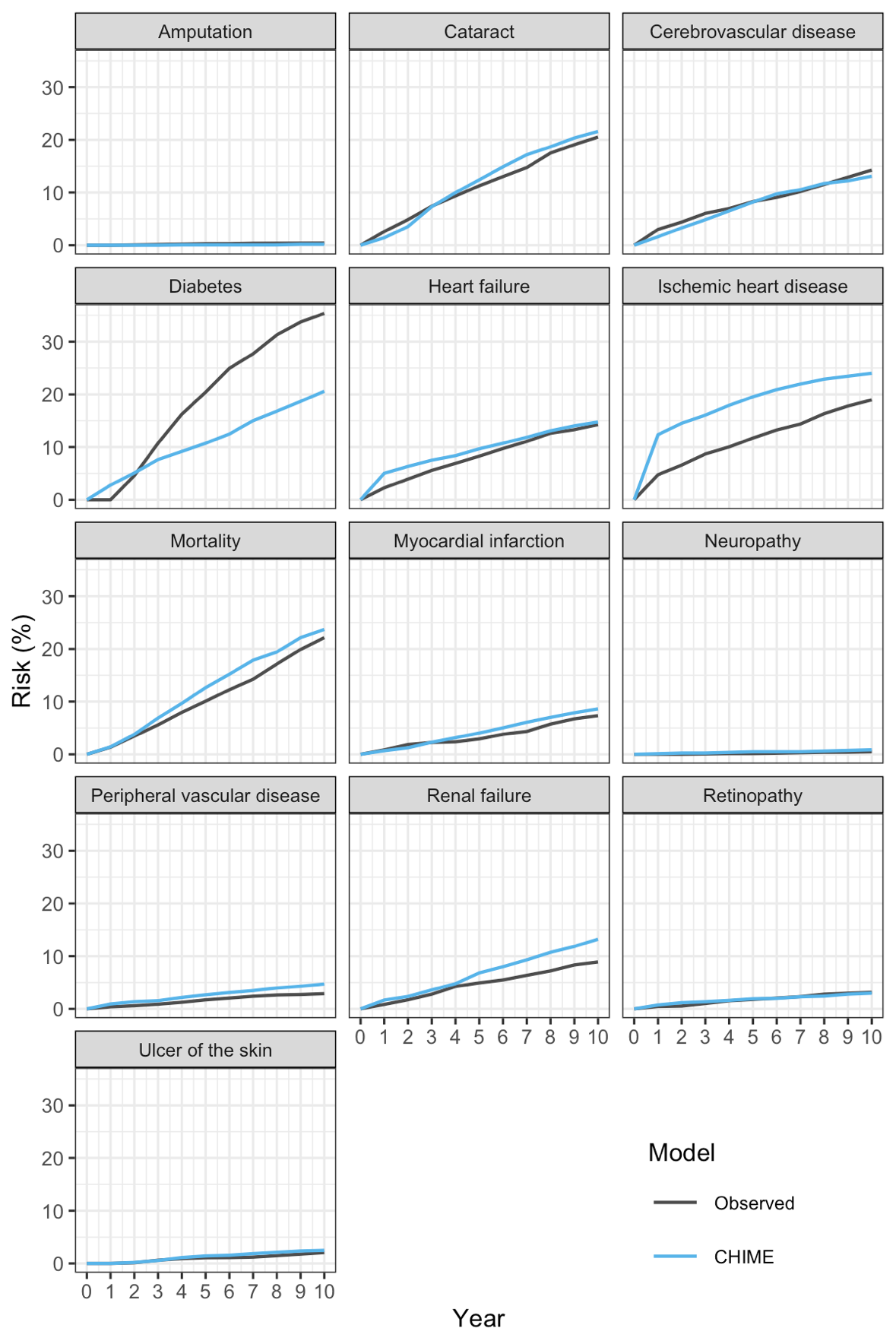


Predicted event rates from CHIME model against the observed events rates over time for CMS participants with prediabetes. CHIME, Chinese Hong Kong Integrated Modelling and Evaluation.
