# Supplementary material for: Development and validation of the CHIME simulation model to assess lifetime health outcomes of prediabetes and type 2 diabetes in Chinese populations: A modeling study
Source: PLoS Med. 2021 Jun 24;18(6):e1003692. doi: 10.1371/journal.pmed.1003692 (PMC8270422; doi:10.1371/journal.pmed.1003692)
Supplement: S3 Fig — See Table 3 for further details of trials. ACCORD, Action to Control Cardiovascular Risk in Diabetes; ACE, Acarbose Cardiovascular Evaluation; ADVANCE, Action in Diabetes and Vascular disease: preterAx and diamicroN-MR Controlled Evaluation; DPP, Diabetes Prevention Program; JDCS, Japan Diabetes Complications Study; J-EDIT, Japan Elderly Diabetes Intervention Trial; JPAD, Japanese Primary Prevention of Atherosclerosis with Aspirin for Diabetes; UKPDS 33, UK Prospective Diabetes Study 33; UKPDS 80, UK Prospective Diabetes Study 80. (PDF) [file pmed.1003692.s004.pdf]

S3 Figure. Convergence plot of trial simulations: all mortality

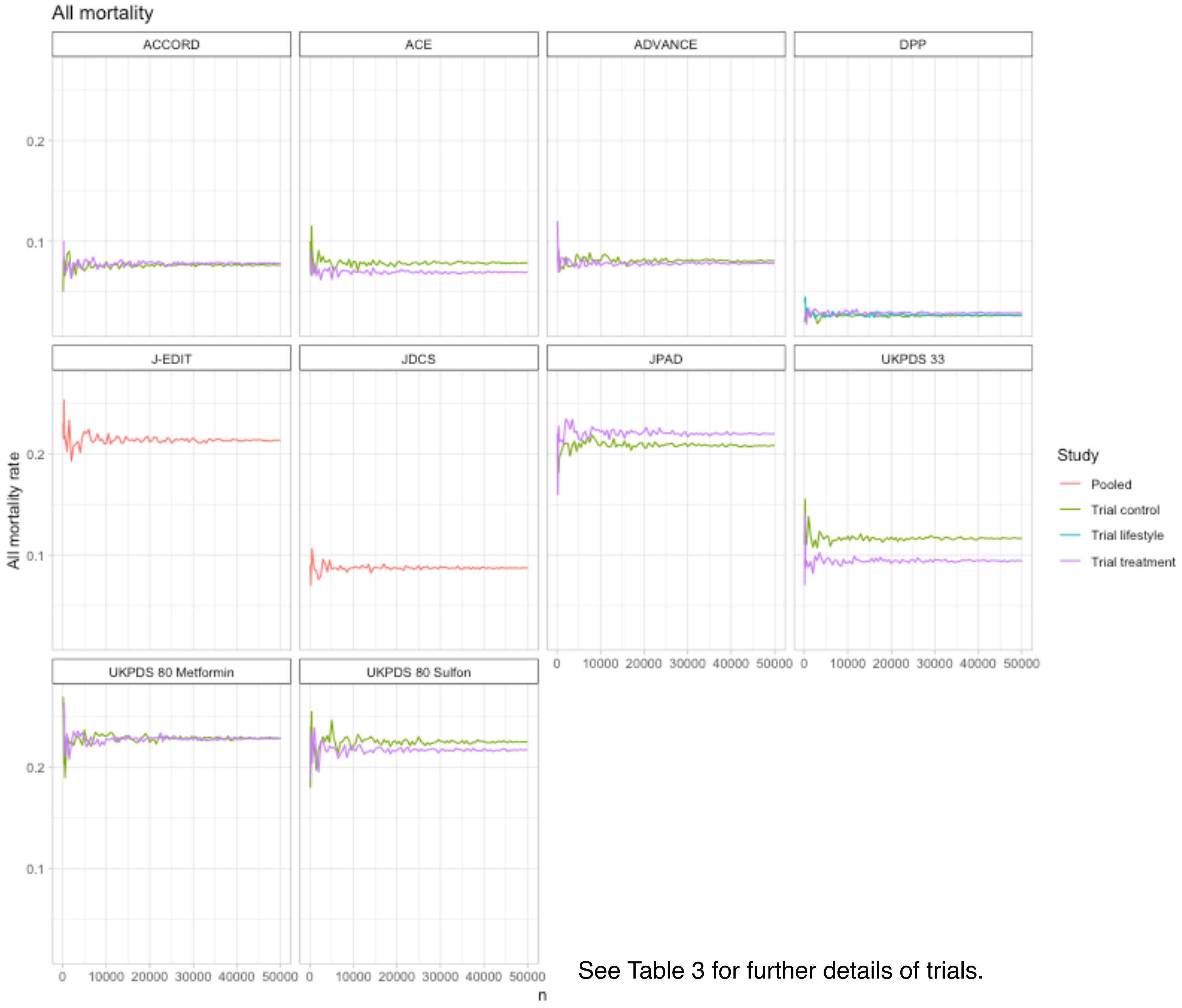


See Table 3 for further details of trials.

S3 Figure. Convergence plot of trial simulations: amputation

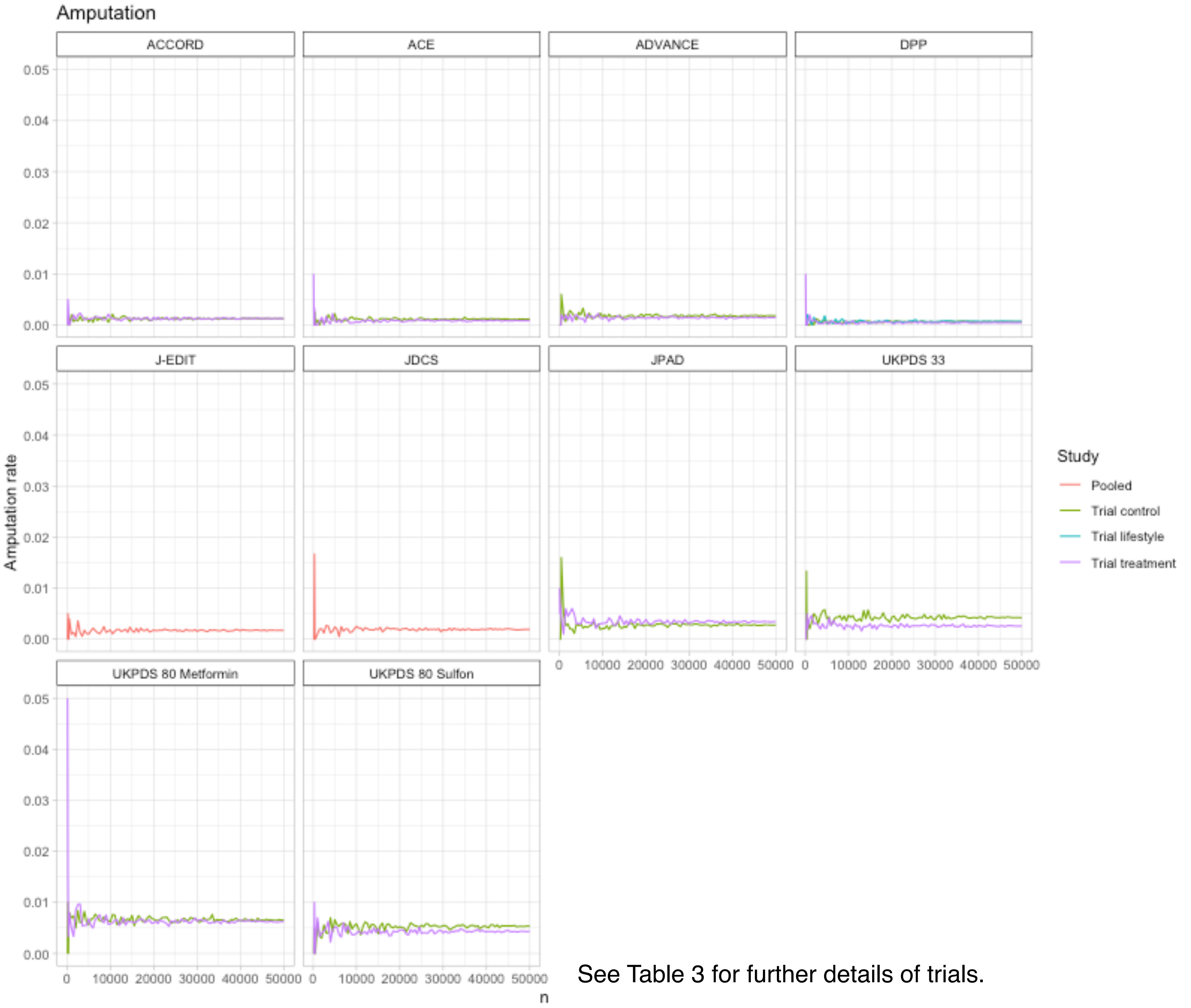


See Table 3 for further details of trials.

S3 Figure. Convergence plot of trial simulations: cataract

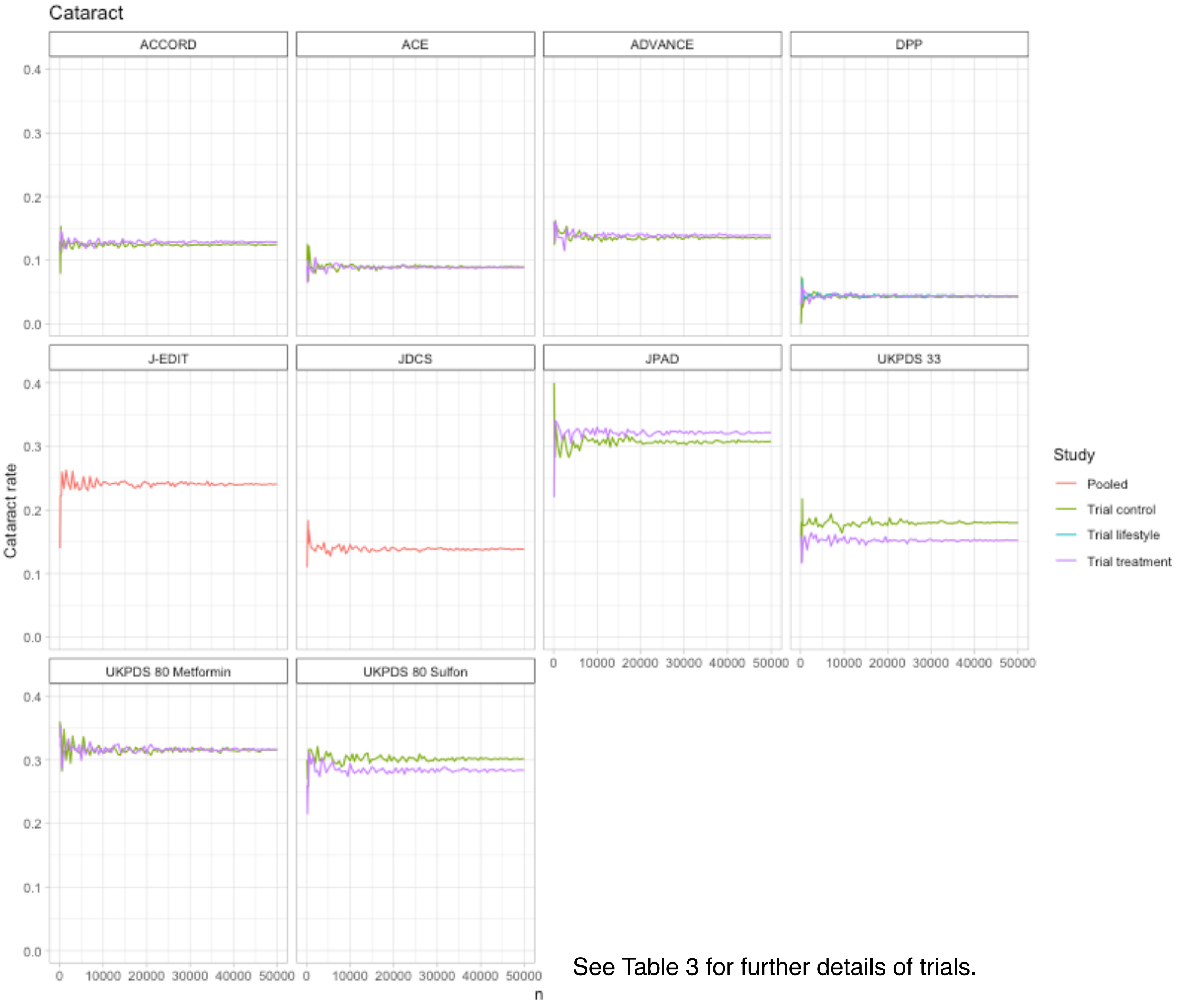


See Table 3 for further details of trials.

S3 Figure. Convergence plot of trial simulations: heart failure

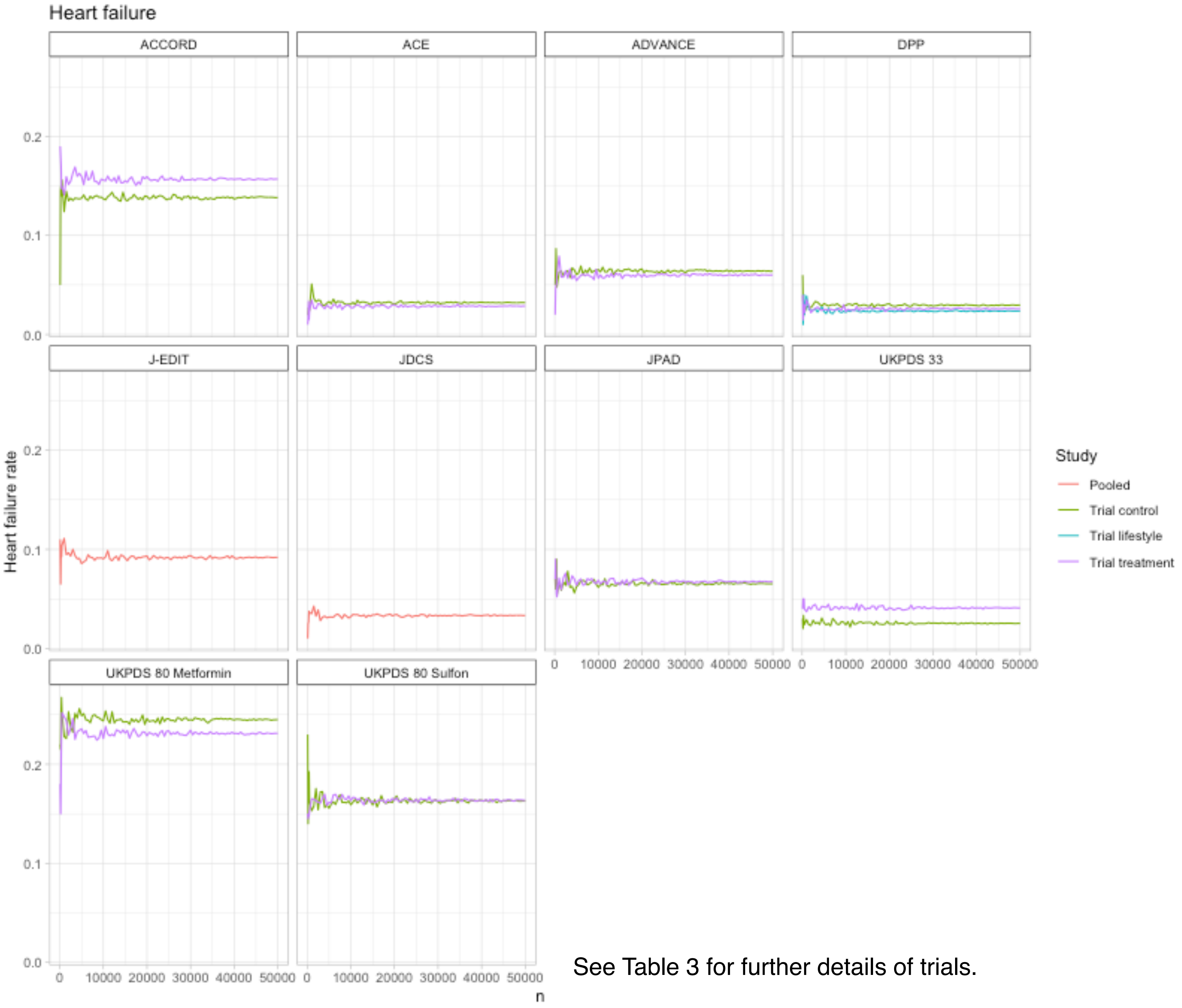


See Table 3 for further details of trials.

S3 Figure. Convergence plot of trial simulations: ischemic heart disease

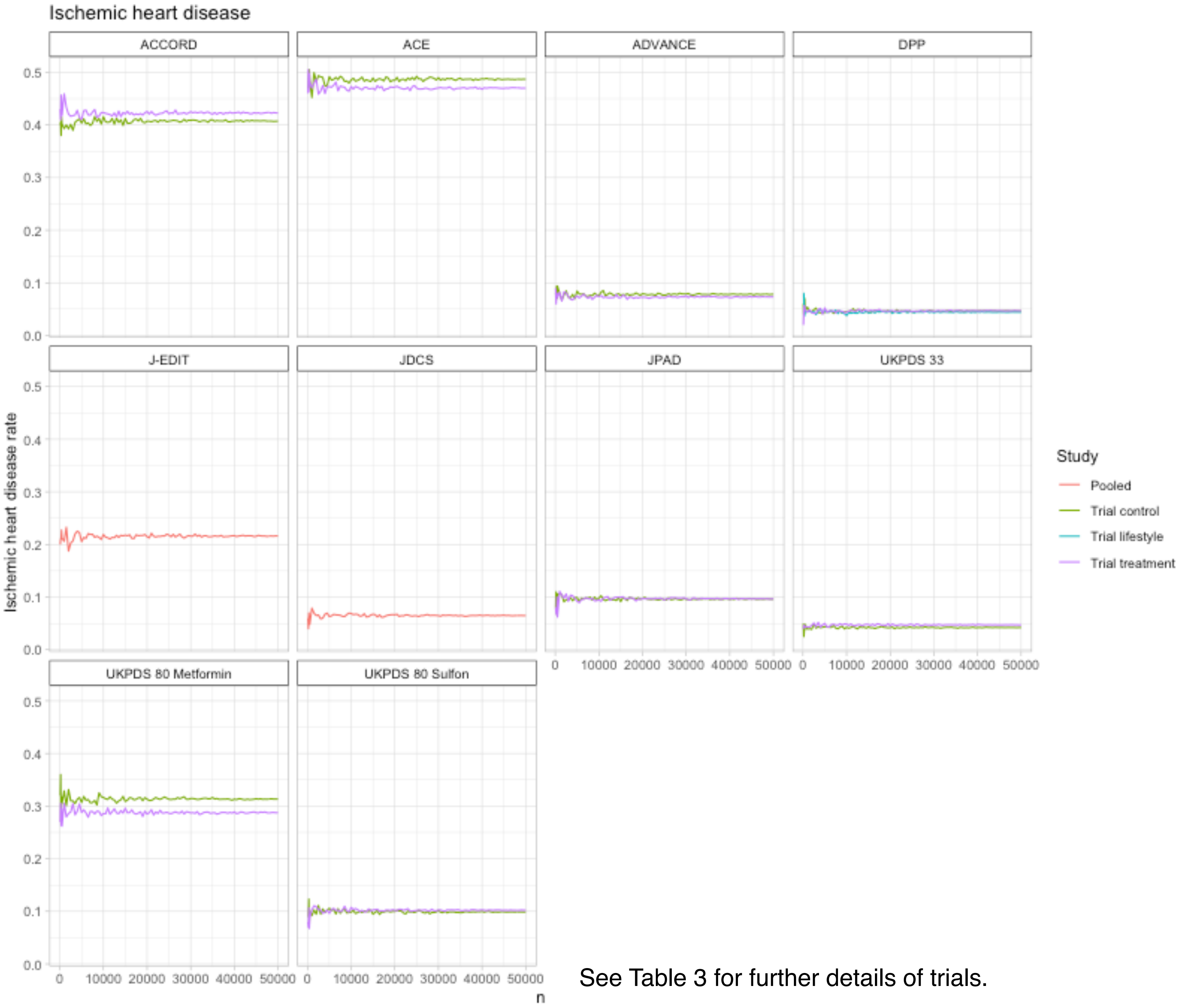


See Table 3 for further details of trials.

S3 Figure. Convergence plot of trial simulations: myocardial infarction

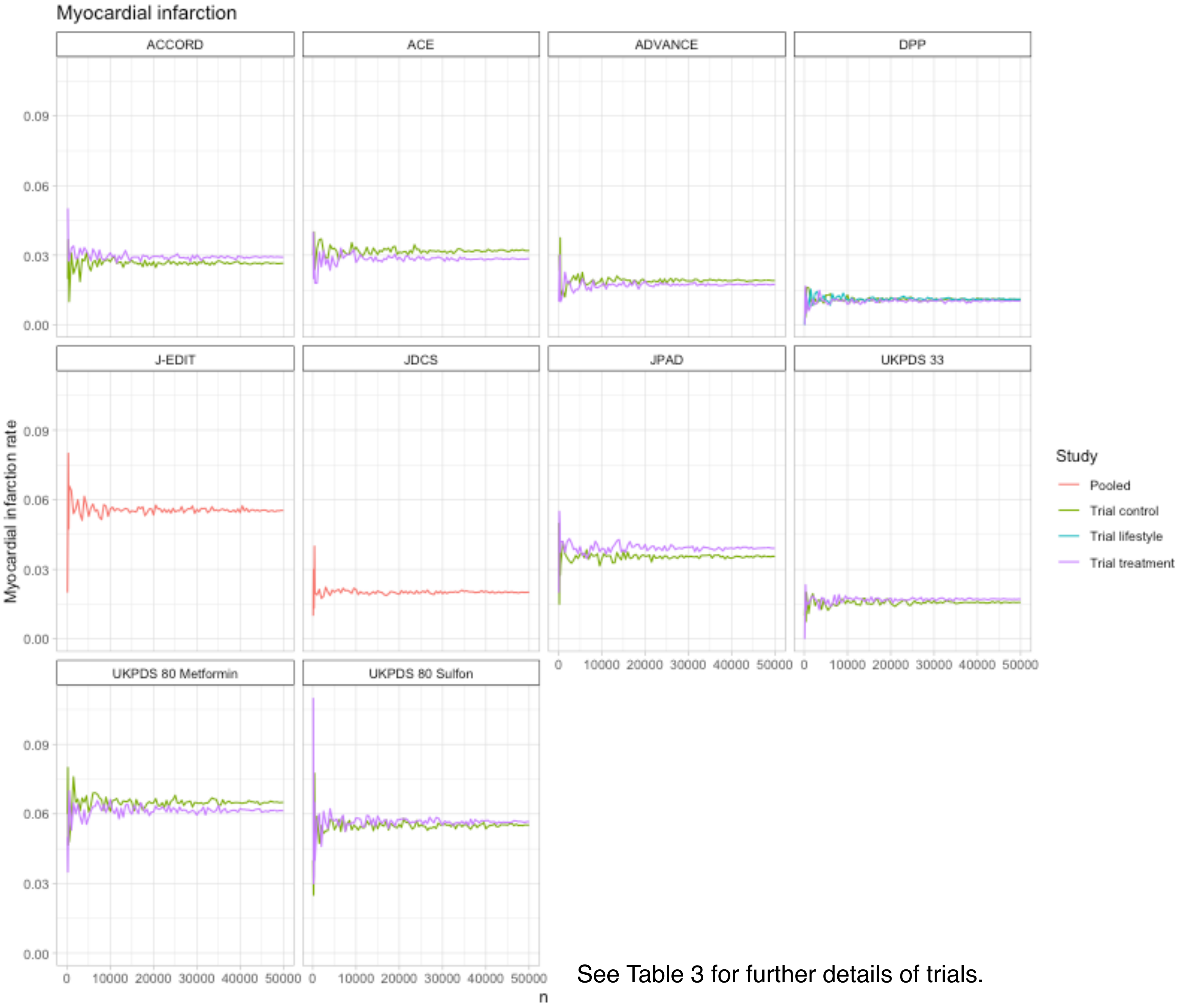


See Table 3 for further details of trials.

S3 Figure. Convergence plot of trial simulations: neuropathy

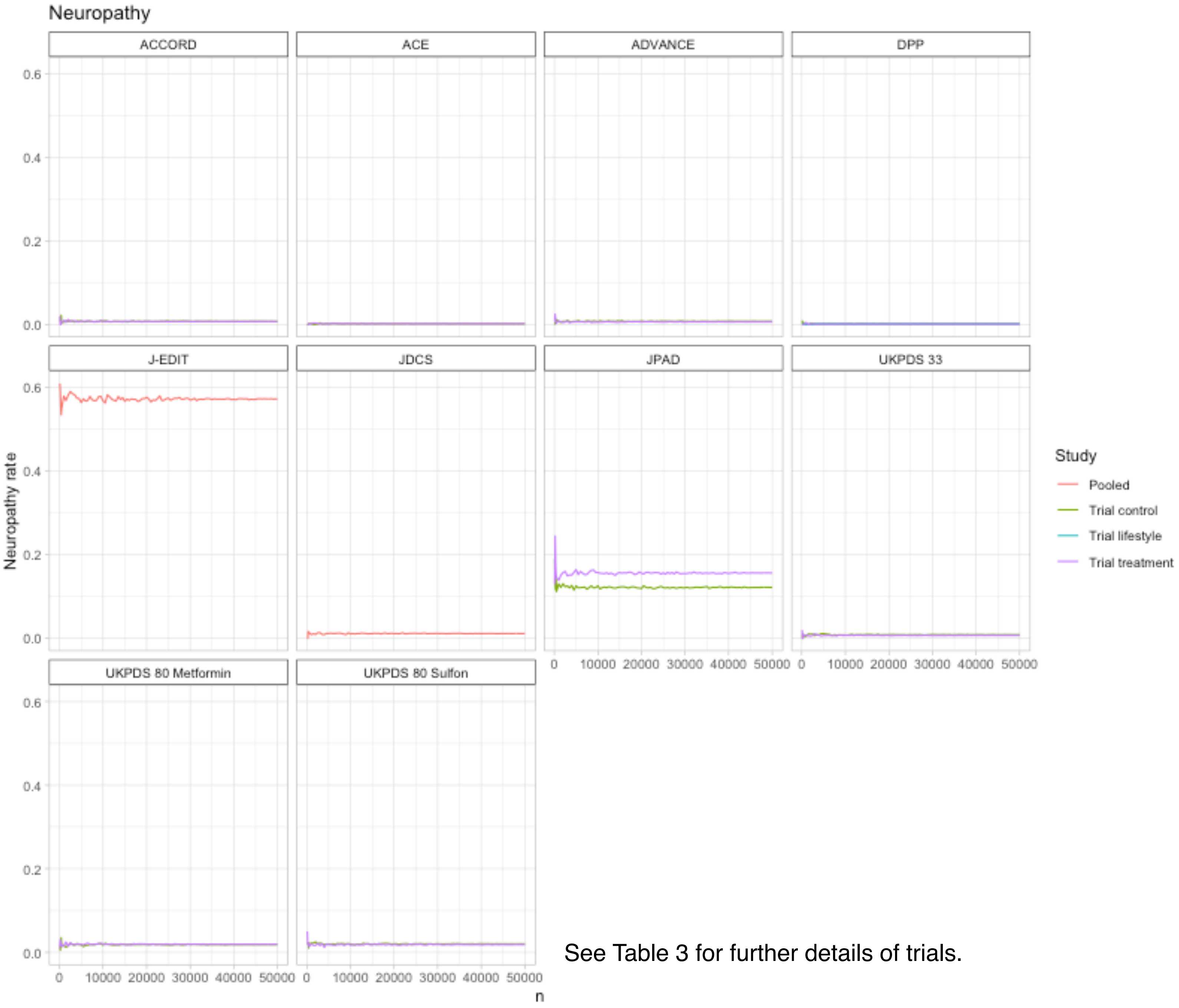


See Table 3 for further details of trials.

S3 Figure. Convergence plot of trial simulations: peripheral vascular disease

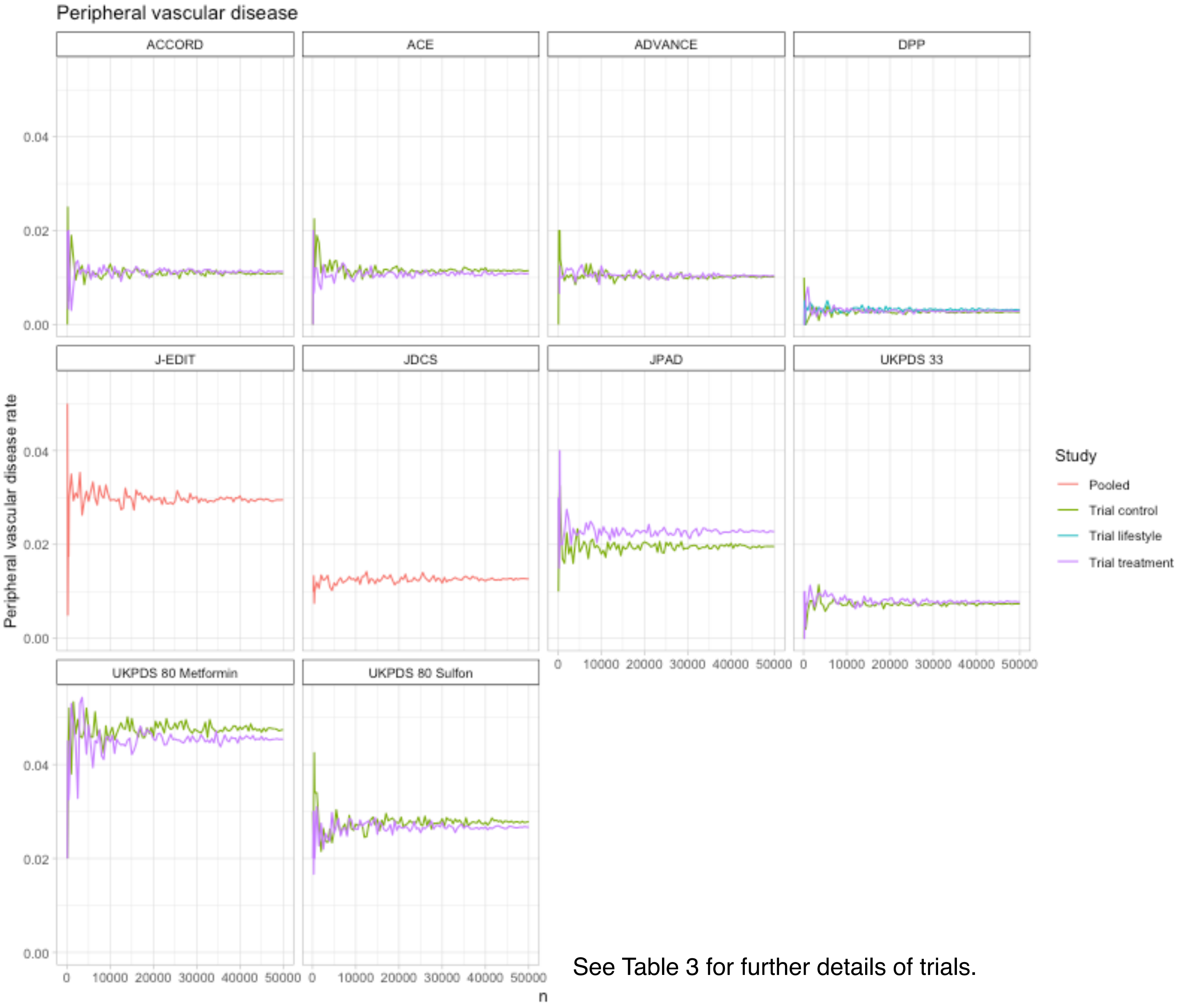


See Table 3 for further details of trials.

S3 Figure. Convergence plot of trial simulations: renal failure

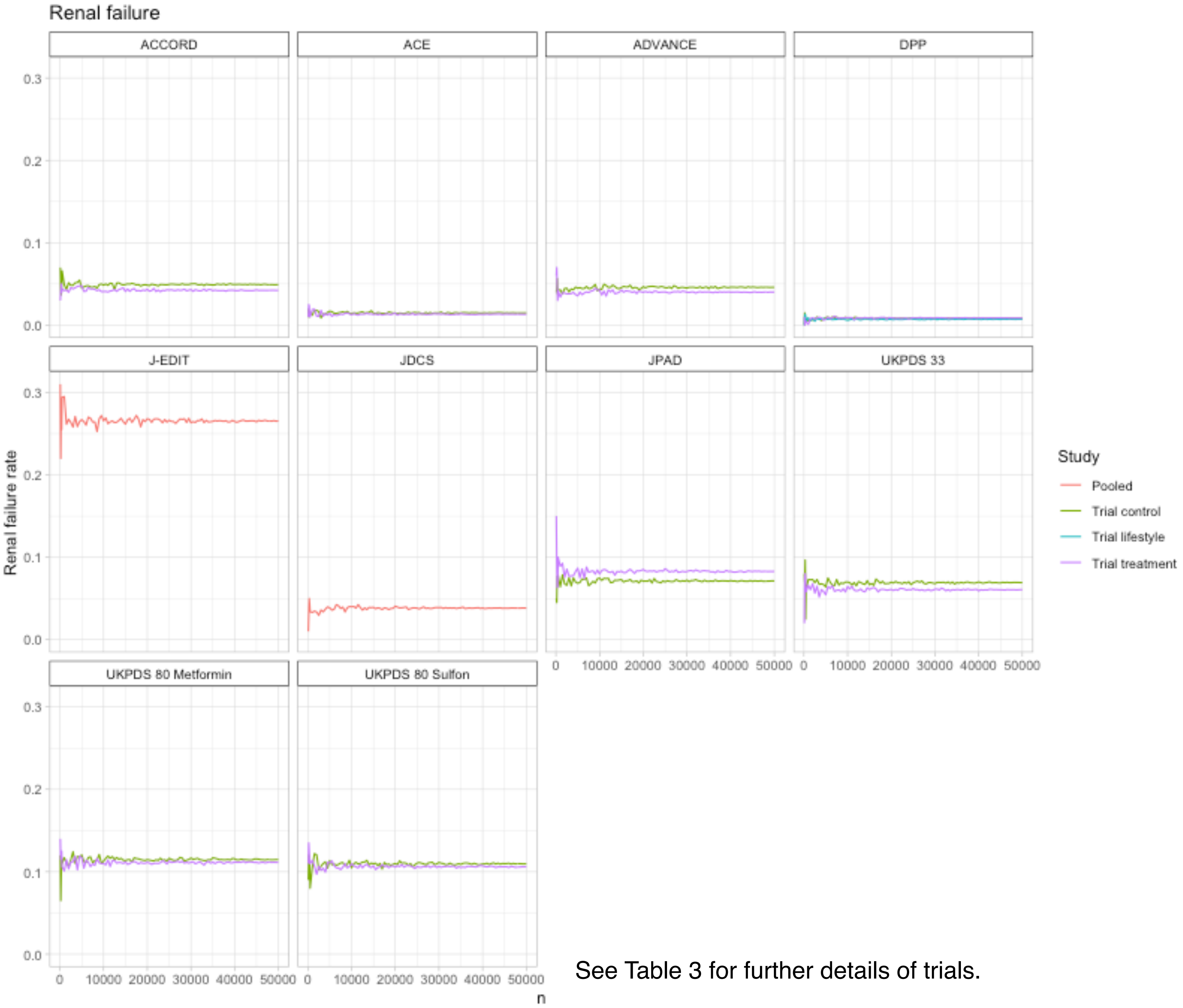


See Table 3 for further details of trials.

S3 Figure. Convergence plot of trial simulations: retinopathy

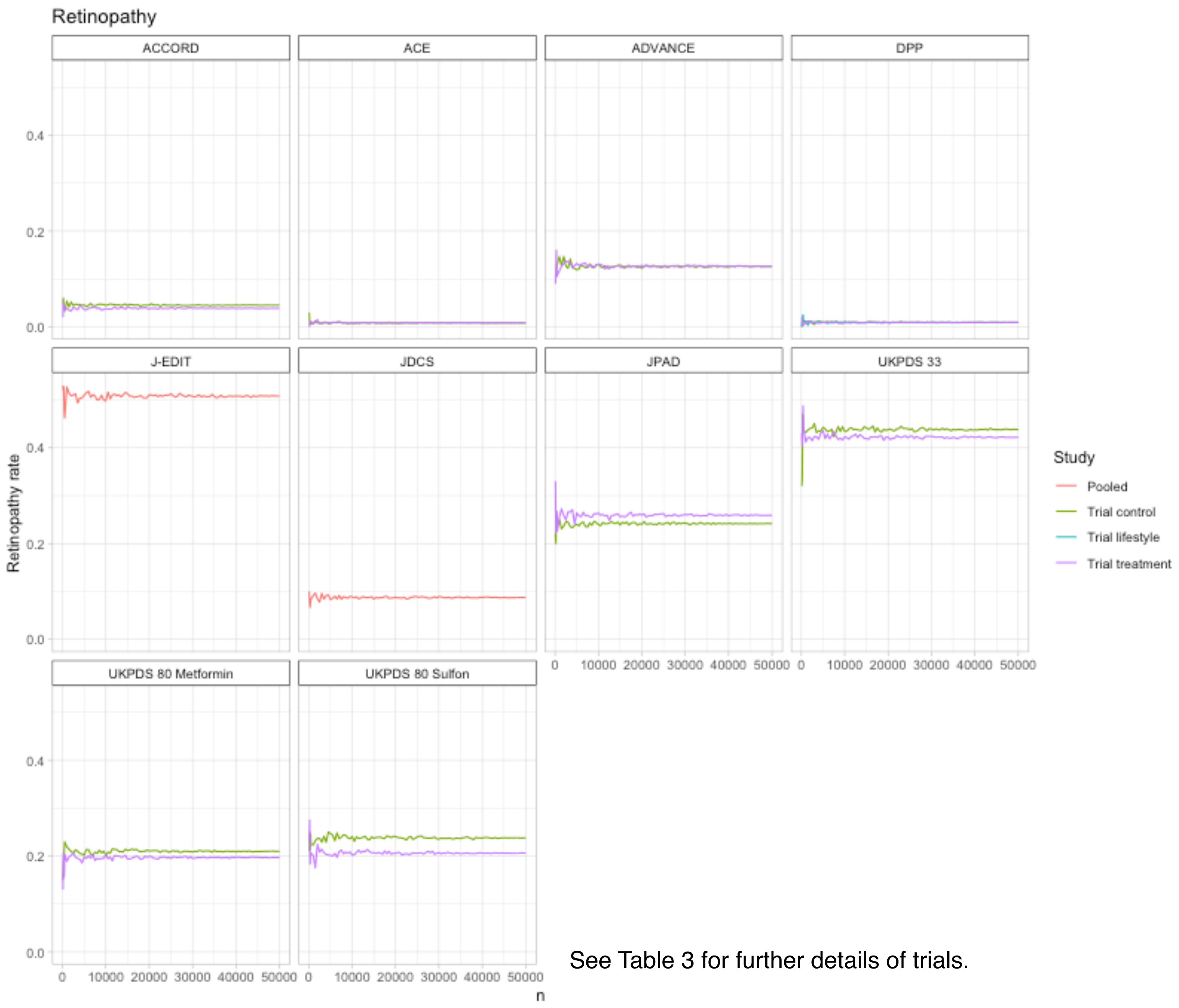


See Table 3 for further details of trials.

S3 Figure. Convergence plot of trial simulations: stroke

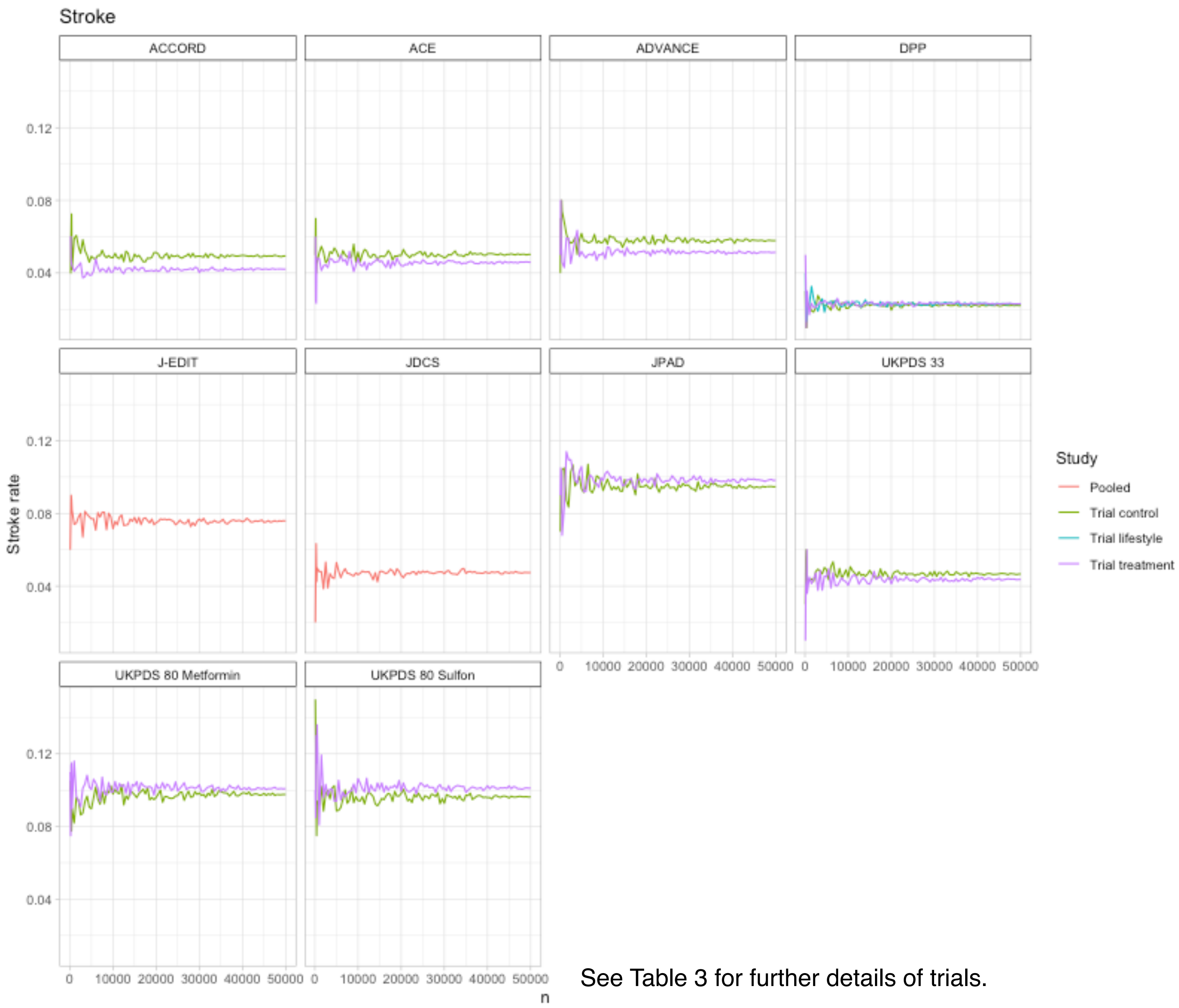


See Table 3 for further details of trials.

S3 Figure. Convergence plot of trial simulations: ulcer of the skin

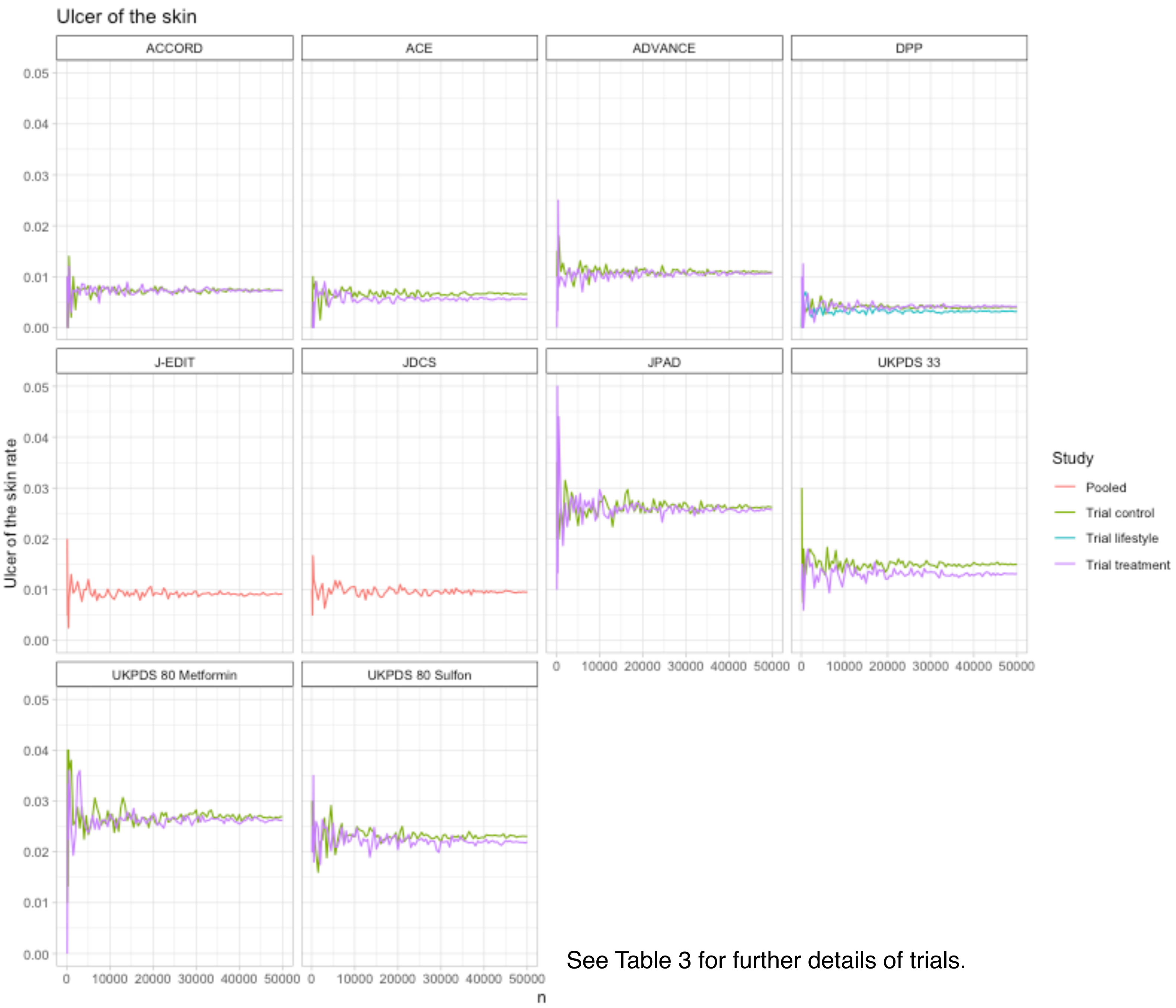


See Table 3 for further details of trials.
