# Supplementary material for: Development and validation of the CHIME simulation model to assess lifetime health outcomes of prediabetes and type 2 diabetes in Chinese populations: A modeling study
Source: PLoS Med. 2021 Jun 24;18(6):e1003692. doi: 10.1371/journal.pmed.1003692 (PMC8270422; doi:10.1371/journal.pmed.1003692)
Supplement: S1 Table — (DOCX) [file pmed.1003692.s005.docx]

## S1 Table. Diabetes outcomes prediction models

| **Model  (Year)** | **Model Development** | **Sample size, n** | **Development cohort (year), location** | **Participants**  **characteristics and**  **ethnicity (%)** | **Model Validation** | **Validation cohort** |
| --- | --- | --- | --- | --- | --- | --- |
| CHIME | Individual-level simulation | 98,715 | HK-CMS  (2006-2018), Hong Kong | Population-based electronic health records.  Ethnicity:  Chinese (92%) | External validation using CHARLS (individual-level data).  Simulated participants from 9 trials against 80 endpoints. | CHARLS, ACE, ACCORD, ADVANCE, CDQDPS, DPP, JDCS, J-DOIT3, J-EDIT, JPAD, UKPDS |
| BRAVO (2018)^1^ | Individual-level simulation | 10,251 | ACCORD  (2001-2005), USA and Canada | Age 40-79 years with type 2 diabetes and glycated haemoglobin > 7.5%, or pre-existing heart disease in USA.  Ethnicity:  Non-Hispanic white: (60.5)  Black: (24.1)  Hispanic: (7.0) | Simulated participants from 3 trials against 28 endpoints. | ASPEN, ADVANCE, CARDS |
| RECODe (2017)^2^ | Cox proportional hazards models | 18,714 | ACCORD  (2001-2009), USA and Canada. | Ethnicity:  Black (19)  Hispanic or Latino (7) | Using individual participant data from trials for validations. | DPPOS, Look ADEAD,  MESA, JHS |
| UKPDS-OM2 (2013)^3^ | Individual-level simulation | 5,102 | UKPDS  (1977-1991), UK | Age 25–65 years and newly diagnosed type 2 diabetes mellitus,  Ethnicity:  White (80)  Asian Indian (10)  Afro-Caribbean (9) | Internal validation. | - |
| ECHO-T2DM (2013)^4^ | Patient-level stochastic microsimulation model | 6315 | UKPDS  (1977-1991), UK  WESDR (1980s), USA | WESDR: diagnosed diabetes at or after 30 years old in southern Wisconsin, USA. | Simulated participants from 6 trials. | CARDS, MICRO-HOPE,  Sasaki et al.,  ADVANCE, ACCORD, ASPEN |
| Cardiff Stochastic Simulation Cost-Utility Model (DiabForecaster) (2006)^5^ | Discrete event stochastic simulation | 1,441 | Eastman DCCT (1997), USA and UKPDS risk engine. | DCCT:  Age 25-74 years with clinically diagnosed non-insulin dependent diabetes in the USA. | Internal validation,  face validation was conducted to ensure correct logical functioning | - |
| CDC-RTI Diabetes Cost-Effectiveness Model (2009)^6^ | Markov model | 5,102 | UKPDS  (1977-1991), UK | Age 25–65 years and newly diagnosed type 2 diabetes mellitus,  Ethnicity:  White (80)  Asian Indian (10)  Afro-Caribbean (9) | Simulated participants from 24 trials/cohort against 47 endpoints. | Eastman et al., Ravid et al., DAIS, Bruno et al., Partanen et al., Humphrey et al., Haffner et al., Lee et al., CARDS, ADOPT, ACCORD, ADVANCE; Gu et al., Sasaki et al., Li et al., Tuomilehto et al., Ramachandran et al., Kosaka et al., Chiasson et al. |
| MDM-TTM (2014) ^7^ | Monte Carlo model | 10,537 | Risk equations from CDC-RTI Diabetes Cost-Effectiveness Model and UKPDS 56 | - | Internal validation | - |
| CDM (2004)^8^ | Combination of Monte Carlo simulation and Markov modelling | 12,920 | UKPDS (1977-1991), UK; DCCT; Framingham Heart Study (1948-1953), USA; WESDR, USA | Ethnicity:  European American (100%). | Simulated participants from 11 trials against 121 endpoints. | DCCT, UKPDS 33, ASPEN, VADT, ADVANCE, ACCORD, ADDITION-Europe, ASCOT, CARDS, UKPDS 80, EDIC |
| MMD CHD (2015)^9^ | Monte Carlo model | 5,102 | UKPDS risk equations | - | External validation using WESDR cohort.  Simulated participants from 5 trials against 16 endpoints. | WESDR,  VADT, ACCORD, ADOPT, ADVANCE, ADDITION |
| SPHR (2014)^10^ | Individual-level simulation | 10,308 | Whitehall II longitudinal cohort (1985-1988), UK | Age 35-55 years civil servants (men and women in London, UK from 1985-88. | Simulated participants against 4 trials/cohort. | UKPDS, EPIC Norfolk cohort, HSE 2003 cohort, ADDITION |
| MICADO (2015)^11^ | Markov-type model | 498,400 | Dutch general practice registries (2003), the Netherlands | Participants with only diabetes and cardiovascular diseases were used from the closed cohort consisting of persons with and without diabetes in 2003. People with diabetes without complications regarding diabetic foot, nephropathy and retinopathy entered into the model for disease simulation. | External validation of 2 outcomes using the Dutch Medical Register.  Simulated participants from 3 trials. | Dutch Medical Register, KP, Swedish National Diabetes Registry, ADVANCE |
| Syreon Diabetes Control Model (2016)^12^ | Markov model | 4,994 | Hungarian sample of the EHIS (2009), Hungry | Age 15 years or older Hungarian inhabitants, Missing parameters were supplemented by 2009–2010 cohort of US NHANES cross-sectional study. | 92 endpoints. | Not reported |
| MMD (2005)^9^ | Semi-Markov model | 1,780 | WESDR cohort (1980-1982), USA | Age 30 years or older diagnosed with diabetes at in southern Wisconsin, USA. | Internal validation. | - |
| EBMI (2008)^13^ | Evidence-based medicine integrator | 8,632 | KP Northwest region health system (2005), USA | Aged 55 years or over with type 2 diabetes. | Simulated participants from 1 trial. | Heart Protection Study |
| Archimedes Diabetes Model (2003)^14^ | Person-by-person, object-by-object simulation | - | NHANES-III (1988-1994), USA.  UKPDS (1997-1991), UK .  DCCT, HOPE, CARE, Lewis, IDNT, IRMA-2, 4S. | NHANES-III: Representative of US civilian, noninstitutionalized population.  Ethnicity:  Non-Hispanic White: (82.4) Non-Hispanic Black: (9.6) Mexican American: (3.0) Other: 199 (5.0) | Simulated participants from 10 trials. | DPP, HPS, MICRO-HOPE, LIPID, HHS, SHEP, LRC-CPPT, MRC hypertension trial, WOSCOPS, VA-HIT |

ACCORD, Action to Control Cardiovascular Risk in Diabetes trial; ACE, Acarbose Cardiovascular Evaluation trial; ADDITION, Anglo-Danish-Dutch Study of Intensive Treatment In People with Screen Detected Diabetes in Primary Care; ADOPT, A Diabetes Outcome Progression trial; ADVANCE, Action in Diabetes and Vascular Disease: Preterax and Diamicron Modified Release Controlled Evaluation trial; ASCOT, Anglo-Scandinavian Cardiac Outcomes Trial-Blood Pressure Lowering Arm; ASPEN, Atorvastatin Study for Prevention of Coronary Heart Disease Endpoints in Non-Insulin-Dependent Diabetes Mellitus trial; BRAVO, Building, Relating, Assessing, and Validating Outcomes of diabetes model; CARDS, Collaborative Atorvastatin Diabetes Study; CARE, Cholesterol and Recurrent Events; CDM, IMS-CORE Diabetes Model; CDQDPS, China Da Qing Diabetes Prevention Study; CHIME, Chinese Hong Kong Integrated Modeling and Evaluation of Outcomes for Prediabetes and Diabetes; CHARLS, China Health and Retirement Longitudinal Study; DAIS, Diabetes Atherosclerosis Intervention Study; DCCT, Diabetes Control and Complications Trial; DPP, Diabetes Prevention Programme; DPPOS, Diabetes Prevention Programme Outcomes Study; MEBMI, Evidence-Based Medicine Integrator; ECHO-T2DM, Economic and Health Outcomes Model of T2DM; EDIC, Epidemiology of Diabetes Interventions and Complications; EHIS, European Health Interview Survey; EPIC, European Prospective Investigation into Cancer; HHS, Helsinki Heart Study; HK-CMS, Hong Kong Clinical Management System; HOPE, Heart Outcomes Prevention Evaluation; HPS, Heart Protection Study; HSE, Health Survey for England; IDNT, Irbesartan Diabetic Nephropathy Trial; IRMA-2, Irbesartan in Microalbuminuria Type 2 Diabetic Nephropathy trial; JDCS, Japan Diabetes Complications Study; J-DOIT3, Japan Diabetes Optimal Treatment study for 3 major risk factors of cardiovascular diseases; JHS, the Jackson Heart Study; JPAD, Japanese Primary Prevention of Atheroslerosis with Aspirin for Diabetes Trial; KP, Kaiser Pemanente; Lewis, ACE Inhibitors and Diabetic Nephropathy Trial; LIPID, Long-Term Intervention with Pravastatin in Ischemic Disease trial; Look AHEAD, Action for Health in Diabetes; LRC-CPPT, Lipid Research Clinics Coronary Primary Prevention Trial; MDM-TTM, Medical Decision Modeling - Treatment Transitions Model; MESA, the Multi-Ethnic Study of Atherosclerosis; MICADO, Modelling Integrated Care for Diabetes based on Observational data; MICRO-HOPE, Microalbuminuria, Cardiovascular and Renal Outcomes - Heart Outcomes Prevention Evaluation trial; MMD, Michigan Model for Diabetes; MMD CHD, Michigan Model for CHD in T2DM patients; MRC, Medical Research Council; NHANES, National Health and Nutrition Examination; RECODe, Risk Equations for Complications of type 2 Diabetes; SHEP, Systolic Hypertension in the Elderly Study; SPHR, School for Public Health Research Diabetes Prevention Model; 4S, Scandinavian Simvastatin Survival Study; UKPDS, UK Prospective Diabetes Study; UKPDS-OM2, UK Prospective Diabetes Study Outcomes Model; VA-HIT, Veterans Affairs High-Density Lipoprotein Cholesterol Interventions Trial; VADT, Veterans Affairs Diabetes Trial; WESDR, Wisconsin Epidemiologic Study of Diabetic Retinopathy; WOSCOPS, West of Scotland Coronary Prevention Study.

1. Shao H, Fonseca V, Stoecker C, Liu S, Shi L. Novel Risk Engine for Diabetes Progression and Mortality in USA: Building, Relating, Assessing, and Validating Outcomes (BRAVO). PharmacoEconomics. 2018 Sep;36(9):1125–34.

2. Basu S, Sussman JB, Berkowitz SA, Hayward RA, Yudkin JS. Development and validation of Risk Equations for Complications Of type 2 Diabetes (RECODe) using individual participant data from randomised trials. The Lancet Diabetes & Endocrinology. 2017 Oct 1;5(10):788–98.

3. Hayes AJ, Leal J, Gray AM, Holman RR, Clarke PM. UKPDS Outcomes Model 2: a new version of a model to simulate lifetime health outcomes of patients with type 2 diabetes mellitus using data from the 30 year United Kingdom Prospective Diabetes Study: UKPDS 82. Diabetologia. 2013 Sep 1;56(9):1925–33.

4. Willis M, Asseburg C, He J. Validation of economic and health outcomes simulation model of type 2 diabetes mellitus (ECHO-T2DM). Journal of Medical Economics. 2013 Aug 1;16(8):1007–21.

5. McEwan P, Peters JR, Bergenheim K, Currie CJ. Evaluation of the costs and outcomes from changes in risk factors in type 2 diabetes using the Cardiff stochastic simulation cost-utility model (DiabForecaster). Current Medical Research and Opinion. 2006 Jan 1;22(1):121–9.

6. Hoerger T, Segel J, Zhang P, Sorensen S. Validation of the CDC-RTI Diabetes Cost-Effectiveness Model [Internet]. 0 ed. Research Triangle Park, NC: RTI Press; 2009 Sep [cited 2020 May 18]. Available from: http://www.rti.org/publication/validation-cdc-rti-diabetes-cost-effectiveness-model

7. Smolen HJ, Murphy DR, Gahn JC, Yu X, Curtis BH. The Evaluation of Clinical and Cost Outcomes Associated with Earlier Initiation of Insulin in Patients with Type 2 Diabetes Mellitus. Journal of Managed Care Pharmacy. 2014 Sep;20(9):968–84.

8. McEwan P, Foos V, Palmer JL, Lamotte M, Lloyd A, Grant D. Validation of the IMS CORE Diabetes Model. Value in Health. 2014 Sep;17(6):714–24.

9. Zhou H, Isaman DJM, Messinger S, Brown MB, Klein R, Brandle M, et al. A Computer Simulation Model of Diabetes Progression, Quality of Life, and Cost. Diabetes Care. 2005 Dec 1;28(12):2856–63.

10. Thomas C, Watson P, Squires H, Chilcott J, Brennan A. Validation of the SPHR Diabetes Prevention Model. Value in Health. 2014 Nov;17(7):A556.

11. van der Heijden AAWA, Feenstra TL, Hoogenveen RT, Niessen LW, de Bruijne MC, Dekker JM, et al. Policy evaluation in diabetes prevention and treatment using a population-based macro simulation model: the MICADO model. Diabetic Medicine. 2015 Dec;32(12):1580–7.

12. Zsólyom A, Merész G, Nagyjánosi L, Nagyistók S, Nagy B, Kaló Z, et al. External Validation of the Syreon Diabetes Model. Value in Health. 2013 Nov;16(7):A590.

13. Brown J. Evidence-Based Medicine Integrator (EBMI): A New Simulation Architecture for Chronic Disease. Clinical Medicine & Research. 2008 Dec 1;6(3–4):124–124.

14. Eddy DM, Schlessinger L. Validation of the Archimedes Diabetes Model. Diabetes Care. 2003 Nov 1;26(11):3102–10.
