# Supplementary material for: Development and validation of the CHIME simulation model to assess lifetime health outcomes of prediabetes and type 2 diabetes in Chinese populations: A modeling study
Source: PLoS Med. 2021 Jun 24;18(6):e1003692. doi: 10.1371/journal.pmed.1003692 (PMC8270422; doi:10.1371/journal.pmed.1003692)
Supplement: S2 Table — (DOCX) [file pmed.1003692.s006.docx]

## Table S2. Definition of outcomes used in the model by International Classification of Disease (ICD-9) and International Classification of Primary Care (ICPC-2) coding

| **Outcomes** | **ICD-9** | **ICPC-2** |
| --- | --- | --- |
| Ischemic heart disease | 410-414 | K74-K76 |
| Myocardial infarction | 410, 412 | K75 |
| Cerebrovascular disease | 430-434, 436 | K90-K91 |
| Heart failure^1^ | 398.91, 402.01, 402.11, 402.91, 404.01, 404.03, 404.11, 404.13, 404.91, 404.93, 425.4, 425.9, 428 | K77 |
| Atrial fibrillation | 427.31-427.32 | K78 |
| Neuropathy | 250.6, 356-357 | N94 |
| Cataract | 366 | F92 |
| Retinopathy | 362 | F83 |
| Peripheral vascular diseases | 250.7, 440-441, 443.1-443.2, 443.8-443.9 | - |
| Skin ulcer | 707 | S97 |
| Amputation of lower limb | 895-897, 997.6  Procedure code 84.1 | E878.5  V49.7 |
| Renal failure^1^ | 403.01, 403.11, 403.91, 404.02-404.03, 404.12-404.13, 404.92-404.93, 585-586, 588 | V42  V45.1  V56 |
| Hemodialysis | 458.21  Procedure code 39.95 | - |

ICD-9: International Classification of Diseases Clinical Modification; ICPC-2: International Classification of Primary Care, Second edition.

1. Quan H, Sundararajan V, Halfon P, Fong A, Burnand B, Luthi J-C, et al. Coding Algorithms for Defining Comorbidities in ICD-9-CM and ICD-10 Administrative Data: Medical Care. 2005 Nov;43(11):1130–9.
