# Supplementary material for: Development and validation of the CHIME simulation model to assess lifetime health outcomes of prediabetes and type 2 diabetes in Chinese populations: A modeling study
Source: PLoS Med. 2021 Jun 24;18(6):e1003692. doi: 10.1371/journal.pmed.1003692 (PMC8270422; doi:10.1371/journal.pmed.1003692)
Supplement: S3 Table — (DOCX) [file pmed.1003692.s007.docx]

## Table S3. Missing data at baseline for CMS dataset

|  | N | % |
| --- | --- | --- |
| HbA1c | 755,896 | 49.4 |
| Systolic blood pressure | 731,024 | 47.7 |
| Diastolic blood pressure | 730,606 | 47.7 |
| Triglycerides | 465,981 | 30.4 |
| HDL-cholesterol | 488,352 | 31.9 |
| LDL-cholesterol | 497,117 | 32.5 |
| BMI | 1,123,511 | 73.4 |
| eGFR | 271,357 | 17.7 |
| White cell count | 612,650 | 40.0 |
| Complete cases | 97,628 | 6.33 |

Total people with prediabetes and diabetes identified in the CMS data, n = 1,542,103.
