# Supplementary material for: Development and validation of the CHIME simulation model to assess lifetime health outcomes of prediabetes and type 2 diabetes in Chinese populations: A modeling study
Source: PLoS Med. 2021 Jun 24;18(6):e1003692. doi: 10.1371/journal.pmed.1003692 (PMC8270422; doi:10.1371/journal.pmed.1003692)
Supplement: S4 Table — (DOCX) [file pmed.1003692.s008.docx]

## Table S4. Missing data at follow-up for CHARLS validation cohort

|  | **Diabetes** | | **Prediabetes** | | **All** | |
| --- | --- | --- | --- | --- | --- | --- |
|  | N | % | N | % | N | % |
| Mortality | 72 | 5.97% | 144 | 4.28% | 216 | 4.73% |
| Ischemic heart disease | 168 | 13.93% | 333 | 9.91% | 501 | 10.97% |
| Renal failure | 173 | 14.34% | 350 | 10.41% | 523 | 11.45% |
| Cerebrovascular disease | 316 | 26.20% | 610 | 18.15% | 926 | 20.28% |
| Cataract | 75 | 6.22% | 138 | 4.11% | 213 | 4.66% |
| Diabetes | 149 | 12.35% | 436 | 12.97% | 585 | 12.81% |
