# Supplementary material for: Development and validation of the CHIME simulation model to assess lifetime health outcomes of prediabetes and type 2 diabetes in Chinese populations: A modeling study
Source: PLoS Med. 2021 Jun 24;18(6):e1003692. doi: 10.1371/journal.pmed.1003692 (PMC8270422; doi:10.1371/journal.pmed.1003692)
Supplement: S5 Table — (DOCX) [file pmed.1003692.s009.docx]

## Table S5. Internal validation of CHIME prediction models on CMS cohort 2006 to 2017 (diabetes and prediabetes)

| **Model** | **Events  (n)** | **Follow-up (person-years)** | **Rate  (per 100,000 person-years)** | **c-statistic^‡^** | **(95% CI)** | **Brier score** | **(95% CI)** | **Distribution** |
| --- | --- | --- | --- | --- | --- | --- | --- | --- |
| Mortality | 9,878 | 397,617 | 2,484.30 | 0.836 | (0.831, 0.838) | 0.098 | (0.097, 0.099) | Log-logistic |
| Myocardial infarction | 2,270 | 392,119 | 578.91 | 0.806 | (0.799, 0.816) | 0.024 | (0.023, 0.025) | Log-normal |
| Ischemic heart disease | 4,265 | 368,246 | 1,158.19 | 0.729 | (0.720, 0.736) | 0.044 | (0.043, 0.045) | Log-normal |
| Heart failure | 3,237 | 382,439 | 846.41 | 0.840 | (0.834, 0.847) | 0.034 | (0.033, 0.035) | Log-logistic |
| Cerebrovascular disease | 4,363 | 382,132 | 1,141.75 | 0.758 | (0.752, 0.765) | 0.045 | (0.044, 0.046) | Log-normal |
| Peripheral vascular disease | 865 | 393,719 | 219.70 | 0.798 | (0.783, 0.811) | 0.009 | (0.008, 0.009) | Log-normal |
| Neuropathy | 457 | 395,473 | 115.56 | 0.801 | (0.778, 0.819) | 0.005 | (0.004, 0.005) | Weibull |
| Amputation | 242 | 396,878 | 60.98 | 0.869 | (0.846, 0.890) | 0.002 | (0.002, 0.003) | Log-normal |
| Ulcer of skin | 995 | 395,374 | 251.26 | 0.829 | (0.812, 0.840) | 0.010 | (0.010, 0.011) | Log-normal |
| Renal failure | 2,314 | 389,957 | 593.40 | 0.873 | (0.865, 0.881) | 0.026 | (0.025, 0.026) | Log-logistic |
| Cataract | 7,010 | 373,473 | 1,876.98 | 0.775 | (0.769, 0.779) | 0.075 | (0.074, 0.076) | Log-normal |
| Retinopathy | 2,802 | 384,035 | 729.62 | 0.783 | (0.776, 0.793) | 0.028 | (0.027, 0.029) | Log-normal |
| Diabetes mellitus* | 6,078 | 171,458 | 3,544.90 | 0.743 | (0.736, 0.749) | 0.120 | (0.118, 0.121) | Log-normal |

CI, Confidence Interval. *Prediabetes participants only. ^‡^ Optimism bias corrected
