# Supplementary material for: Development and validation of the CHIME simulation model to assess lifetime health outcomes of prediabetes and type 2 diabetes in Chinese populations: A modeling study
Source: PLoS Med. 2021 Jun 24;18(6):e1003692. doi: 10.1371/journal.pmed.1003692 (PMC8270422; doi:10.1371/journal.pmed.1003692)
Supplement: S6 Table — (DOCX) [file pmed.1003692.s010.docx]

## Table S6. Coefficients of predictors for CHIME biomarkers

|  | HbA1c | SE | SBP | SE | DBP | SE | LDL | SE | HDL | SE | TG | SE | BMI | SE |
| --- | --- | --- | --- | --- | --- | --- | --- | --- | --- | --- | --- | --- | --- | --- |
| Predictor |  |  |  |  |  |  |  |  |  |  |  |  |  |  |
| Female | 0.008 | 0.004 | 0.317 | 0.042 | -0.427 | 0.025 | 0.057 | 0.003 | 0.018 | 0.001 | 0.031 | 0.003 | -0.004 | 0.007 |
| Duration of diabetes (years) | 0.004 | 0.001 | 0.104 | 0.01 | 0.053 | 0.006 | 0.003 | 0.001 | -0.001 | 0 | 0.004 | 0.001 | 0 | 0.002 |
| Lag1 | 0.631 | 0.003 | 0.518 | 0.002 | 0.564 | 0.002 | 0.529 | 0.002 | 0.592 | 0.002 | 0.459 | 0.002 | 0.756 | 0.003 |
| Lag2 | 0.076 | 0.002 | 0.169 | 0.002 | 0.229 | 0.002 | 0.164 | 0.002 | 0.311 | 0.002 | 0.29 | 0.002 | 0.22 | 0.003 |
| Insulin | 0.308 | 0.008 | - | - | - | - | - | - | - | - | - | - | - | - |
| Non-insulin hypoglycemic agents | 0.19 | 0.006 | - | - | - | - | - | - | - | - | - | - | - | - |
| Anti-hypertensives | - | - | 0.614 | 0.046 | -0.369 | 0.027 | - | - | - | - | - | - | - | - |
| Statins | - | - | - | - | - | - | -0.229 | 0.003 | 0.005 | 0.001 | -0.031 | 0.003 | - | - |
| Diabetes status | 0.158 | 0.011 | -0.937 | 0.058 | -0.453 | 0.035 | -0.062 | 0.004 | -0.014 | 0.001 | 0.011 | 0.005 | -0.007 | 0.01 |
| (Intercept) | 1.526 | 0.022 | 41.397 | 0.253 | 15.835 | 0.126 | 0.831 | 0.008 | 0.139 | 0.003 | 0.319 | 0.007 | 0.587 | 0.027 |
| Root mean square error | 0.72 |  | 9.70 |  | 5.80 |  | 0.53 |  | 0.18 |  | 0.64 |  | 1.20 |  |

SE, standard error; HbA1c, glycosylated hemoglobin type A1c (%); SBP, systolic blood pressure (mmHg); DBP, diastolic blood pressure (mmHg); HDL, HDL cholesterol (mmol/L); LDL, LDL cholesterol (mmol/L); TG, triglycerides (mmol/L), BMI, body mass index (kg/m^2^)
