# Supplementary material for: Development and validation of the CHIME simulation model to assess lifetime health outcomes of prediabetes and type 2 diabetes in Chinese populations: A modeling study
Source: PLoS Med. 2021 Jun 24;18(6):e1003692. doi: 10.1371/journal.pmed.1003692 (PMC8270422; doi:10.1371/journal.pmed.1003692)
Supplement: S7 Table — (DOCX) [file pmed.1003692.s011.docx]

## Table S7. Survival time ratios of predictors in the CHIME risk equations

| Predictor | Mortality | | MI | | IHD | | Heart failure | | Cerebrovascular | | PVD | | Neuropathy | |
| --- | --- | --- | --- | --- | --- | --- | --- | --- | --- | --- | --- | --- | --- | --- |
| Age (years) | 0.504 | (0.490, 0.519) | 0.545 | (0.506, 0.587) | 0.566 | (0.527, 0.609) | 0.378 | (0.350, 0.409) | 0.417 | (0.392, 0.444) | 0.432 | (0.373, 0.501) | - | - |
| Female | 1.65 | (1.592, 1.710) | 1.678 | (1.503, 1.872) | 2.05 | (1.837, 2.288) | 1.194 | (1.081, 1.318) | 1.434 | (1.323, 1.555) | 2.039 | (1.626, 2.559) | 2.533 | (1.967, 3.262) |
| Diabetes status | 0.919 | (0.880, 0.960) | - | - | - | - | 0.744 | (0.660, 0.838) | 0.73 | (0.656, 0.812) | - | - | 0.33 | (0.216, 0.503) |
| Duration of diabetes (years) | 0.861 | (0.799, 0.927) | 0.704 | (0.555, 0.894) | 0.441 | (0.339, 0.575) | 0.663 | (0.546, 0.805) | 0.64 | (0.512, 0.798) | 0.434 | (0.274, 0.687) | 0.368 | (0.251, 0.542) |
| Current smoker | 0.837 | (0.797, 0.879) | 0.702 | (0.608, 0.809) | 0.892 | (0.769, 1.036) | 0.799 | (0.690, 0.925) | - | - | 0.448 | (0.336, 0.596) | - | - |
| Past smoker | 1.02 | (0.983, 1.058) | 0.858 | (0.767, 0.960) | 0.738 | (0.658, 0.826) | 0.812 | (0.733, 0.899) | - | - | 0.517 | (0.412, 0.649) | - | - |
| BMI | 1.282 | (1.256, 1.308) | 1.206 | (1.128, 1.290) | - | - | 0.838 | (0.789, 0.891) | 1.14 | (1.078, 1.206) | 1.423 | (1.245, 1.625) | 1.47 | (1.260, 1.716) |
| HbA1c | 0.971 | (0.954, 0.989) | 0.888 | (0.850, 0.927) | - | - | 0.911 | (0.866, 0.958) | 0.99 | (0.943, 1.038) | 0.837 | (0.767, 0.913) | 0.718 | (0.612, 0.842) |
| SBP | 1.19 | (1.128, 1.254) | 0.727 | (0.615, 0.859) | 0.719 | (0.606, 0.853) | 0.944 | (0.811, 1.098) | 0.583 | (0.503, 0.676) | - | - | 0.723 | (0.519, 1.007) |
| DBP | 0.785 | (0.731, 0.844) | 0.768 | (0.628, 0.939) | 0.84 | (0.686, 1.029) | 0.672 | (0.558, 0.810) | 0.616 | (0.525, 0.723) | - | - | - | - |
| HDL | 1.089 | (1.061, 1.118) | 1.406 | (1.301, 1.521) | 1.505 | (1.389, 1.631) | 1.309 | (1.216, 1.410) | 1.281 | (1.197, 1.371) | 1.354 | (1.154, 1.588) | - | - |
| LDL | 0.935 | (0.908, 0.963) | - | - | 1.05 | (0.962, 1.145) | - | - | - | - | - | - | - | - |
| TG | - | - | - | - | - | - | 1.087 | (1.057, 1.118) | - | - | - | - | - | - |
| Hemoglobin | 1.212 | (1.180, 1.244) | 1.286 | (1.191, 1.388) | 1.258 | (1.171, 1.352) | 1.264 | (1.173, 1.362) | 1.216 | (1.144, 1.293) | 1.442 | (1.235, 1.683) | 1.492 | (1.236, 1.801) |
| WBC | 0.827 | (0.812, 0.843) | 0.687 | (0.648, 0.727) | 0.723 | (0.681, 0.769) | 0.792 | (0.751, 0.834) | 0.753 | (0.716, 0.792) | 0.697 | (0.620, 0.784) | 0.903 | (0.776, 1.052) |
| eGFR |  |  |  |  |  |  |  |  |  |  |  |  |  |  |
| normal | ref |  | ref |  | ref |  | ref |  |  |  | ref |  | ref |  |
| mild | 1.151 | (1.112, 1.192) | 0.82 | (0.737, 0.912) | 0.777 | (0.702, 0.859) | 0.691 | (0.621, 0.768) | - | - | 0.856 | (0.690, 1.062) | 0.981 | (0.757, 1.272) |
| mild- moderate | 1.024 | (0.973, 1.076) | 0.541 | (0.463, 0.633) | 0.515 | (0.436, 0.608) | 0.405 | (0.353, 0.465) | - | - | 0.417 | (0.305, 0.571) | 0.534 | (0.363, 0.785) |
| moderate-severe | 0.927 | (0.869, 0.989) | 0.401 | (0.328, 0.490) | 0.409 | (0.325, 0.514) | 0.323 | (0.272, 0.383) | - | - | 0.398 | (0.263, 0.602) | 0.429 | (0.264, 0.699) |
| severe | 0.746 | (0.685, 0.814) | 0.339 | (0.255, 0.451) | 0.319 | (0.229, 0.444) | 0.246 | (0.195, 0.309) | - | - | 0.259 | (0.147, 0.454) | 0.163 | (0.097, 0.275) |
| renal failure | 0.680 | (0.592, 0.779) | 0.215 | (0.139, 0.332) | 0.294 | (0.173, 0.502) | 0.222 | (0.155, 0.320) | - | - | 0.130 | (0.058, 0.294) | 0.136 | (0.063, 0.297) |
| Insulin | - | - | - | - | - | - | 0.66 | (0.520, 0.836) | - | - | - | - | - | - |
| Non-insulin hypoglycemic agents | 0.785 | (0.750, 0.821) | - | - | - | - | 0.847 | (0.749, 0.958) | - | - | - | - | - | - |
| Anti-hypertensives | 0.929 | (0.897, 0.963) | - | - | 0.809 | (0.736, 0.890) | 0.817 | (0.740, 0.902) | - | - | - | - | - | - |
| Statins | 1.06 | (1.013, 1.108) | - | - | 0.536 | (0.472, 0.609) | - | - | - | - | - | - | - | - |
| AF | 0.764 | (0.718, 0.812) | - | - | - | - | 0.319 | (0.275, 0.369) | 0.492 | (0.420, 0.577) | - | - | - | - |
| MI | - | - | 0.578 | (0.443, 0.755) | - | - | 0.696 | (0.543, 0.893) | - | - | - | - | - | - |
| IHD | 0.923 | (0.872, 0.976) | 0.543 | (0.439, 0.671) | - | - | 0.597 | (0.495, 0.721) | - | - | - | - | - | - |
| HF | 0.717 | (0.673, 0.764) | 0.627 | (0.514, 0.764) | 0.5 | (0.390, 0.640) | - | - | - | - | - | - | - | - |
| Cerebrovascular | 0.864 | (0.825, 0.905) | - | - | 1.792 | (1.516, 2.119) | - | - | 0.267 | (0.240, 0.298) | - | - | - | - |
| PVD | 0.844 | (0.743, 0.959) | - | - | - | - | - | - | - | - | - | - | - | - |
| Amputation | - | - | - | - | - | - | - | - | - | - | 0.073 | (0.016, 0.338) | - | - |
| Ulcer of skin | - | - | - | - | - | - | - | - | - | - | 0.253 | (0.107, 0.597) | - | - |

| Predictor | Amputation | | Ulcer of skin | | Renal failure | | Cataract | | Retinopathy | | Diabetes | |
| --- | --- | --- | --- | --- | --- | --- | --- | --- | --- | --- | --- | --- |
| Age (years) | 0.674 | (0.571, 0.795) | 0.513 | (0.470, 0.560) | 0.738 | (0.697, 0.781) | 0.391 | (0.374, 0.409) | - | - | - | - |
| Female | 2.461 | (1.839, 3.294) | 1.634 | (1.439, 1.855) | 2.948 | (2.685, 3.237) | - | - | 1.721 | (1.548, 1.913) | - | - |
| Diabetes status | - | - | 0.575 | (0.477, 0.693) | 0.773 | (0.694, 0.861) | - | - | 0.501 | (0.422, 0.594) | - | - |
| Duration of diabetes (years) | - | - | 0.857 | (0.632, 1.163) | 0.436 | (0.376, 0.505) | 0.83 | (0.719, 0.959) | - | - | - | - |
| Current smoker | - | - | - | - | 1.057 | (0.923, 1.211) | 1.053 | (0.966, 1.149) | - | - | 0.91 | (0.834, 0.993) |
| Past smoker | - | - | - | - | 0.911 | (0.832, 0.997) | 0.873 | (0.826, 0.924) | - | - | 0.84 | (0.789, 0.895) |
| BMI | - | - | 1.154 | (1.062, 1.254) | - | - | - | - | 1.309 | (1.215, 1.409) | 0.94 | (0.903, 0.978) |
| HbA1c | 0.516 | (0.436, 0.610) | 0.861 | (0.798, 0.929) | 0.913 | (0.873, 0.955) | 0.874 | (0.853, 0.895) | 0.57 | (0.528, 0.615) | 0.127 | (0.117, 0.138) |
| SBP | 0.693 | (0.457, 1.052) | - | - | 0.499 | (0.430, 0.579) | 0.844 | (0.772, 0.922) | 0.467 | (0.401, 0.544) | 0.756 | (0.698, 0.819) |
| DBP | - | - | 0.663 | (0.516, 0.853) | 0.903 | (0.765, 1.066) | 1.12 | (0.991, 1.265) | - | - | - | - |
| HDL | - | - | - | - | - | - | - | - | 0.886 | (0.810, 0.970) | 1.098 | (1.039, 1.160) |
| LDL | - | - | - | - | 0.827 | (0.772, 0.887) | - | - | - | - | 1.057 | (1.008, 1.109) |
| TG | - | - | - | - | 0.879 | (0.860, 0.898) | - | - | - | - | 0.93 | (0.914, 0.946) |
| Hemoglobin | 2.136 | (1.639, 2.782) | 1.574 | (1.404, 1.764) | 1.72 | (1.590, 1.862) | 1.153 | (1.108, 1.200) | 1.454 | (1.346, 1.570) | 0.93 | (0.894, 0.967) |
| WBC | 0.587 | (0.497, 0.693) | 0.713 | (0.661, 0.769) | 0.925 | (0.882, 0.971) | 0.959 | (0.927, 0.992) | - | - | 0.807 | (0.776, 0.840) |
| eGFR |  |  |  |  |  |  |  |  |  |  |  |  |
| normal |  |  |  |  |  |  |  |  | ref |  |  |  |
| mild | - | - | - | - | - | - | - | - | 0.957 | (0.862, 1.062) | - | - |
| mild- moderate | - | - | - | - | - | - | - | - | 0.707 | (0.583, 0.857) | - | - |
| moderate-severe | - | - | - | - | - | - | - | - | 0.548 | (0.419, 0.717) | - | - |
| severe | - | - | - | - | - | - | - | - | 0.349 | (0.240, 0.508) | - | - |
| renal failure | - | - | - | - | - | - | - | - | 0.164 | (0.094, 0.286) | - | - |
| Non-insulin hypoglycemic agents | - | - | 0.723 | (0.614, 0.851) | - | - | - | - | 0.691 | (0.602, 0.793) | - | - |
| Anti-hypertensives | - | - | - | - | 0.762 | (0.700, 0.830) | 1.086 | (1.030, 1.144) | 1.566 | (1.398, 1.754) | - | - |
| Statins | - | - | - | - | - | - | - | - | - | - | 1.238 | (1.157, 1.326) |
| MI | - | - | - | - | - | - | - | - | - | - | 0.814 | (0.710, 0.935) |
| IHD | - | - | - | - | - | - | - | - | 1.633 | (1.213, 2.197) | - | - |
| HF | - | - | - | - | 0.511 | (0.442, 0.590) | - | - | - | - | 0.788 | (0.685, 0.907) |
| Cerebrovascular disease | - | - | 0.707 | (0.582, 0.859) | 0.835 | (0.741, 0.941) | - | - | - | - | - | - |
| PVD | - | - | - | - | 0.73 | (0.547, 0.973) | - | - | - | - | 0.601 | (0.462, 0.781) |
| Amputation | 0.03 | (0.010, 0.089) | 0.153 | (0.075, 0.315) | - | - | - | - | - | - | - | - |
| Retinopathy | - | - | - | - | 0.613 | (0.490, 0.766) | 0.597 | (0.507, 0.703) | - | - | - | - |
| Cataract | - | - | - | - | 0.849 | (0.758, 0.952) | 0.373 | (0.348, 0.400) | 0.745 | (0.612, 0.906) | - | - |
| Ulcer of skin | 0.146 | (0.060, 0.354) | 0.112 | (0.073, 0.172) | - | - | - | - | - | - | - | - |

(95% confidence interval); BMI, body mass index (kg/m^2^); HbA1c, glycosylated hemoglobin type A1c (%); SBP, systolic blood pressure (mmHg); DBP, diastolic blood pressure (mmHg); HDL, HDL cholesterol (mmol/L); LDL, LDL cholesterol (mmol/L); TG, triglycerides (mmol/L); eGFR, estimated glomerular filtration rate (mL/min/1.73m^2^); Hemoglobin (g/L); WBC, white blood cell count (x10^9^/L); HF, heart failure; AF, atrial fibrillation; IHD, ischemic heart disease; MI, myocardial infarction; PVD, peripheral vascular diseases; RF, renal failure; DM, diabetes mellitus.

Survival time ratio (where <1 indicated shorter survival time). All effects are centered on a common scale to allow the relative strength of various predictors to be judged. Survival time ratios vary continuous predictors from the first to the third quartile.^[[1]](#footnote-2)^

1. Harrell FE. Regression Modeling Strategies. 2nd ed. Cham: Springer International Publishing; 2015. (Springer Series in Statistics). [↑](#footnote-ref-2)
