# Supplementary material for: Development and validation of the CHIME simulation model to assess lifetime health outcomes of prediabetes and type 2 diabetes in Chinese populations: A modeling study
Source: PLoS Med. 2021 Jun 24;18(6):e1003692. doi: 10.1371/journal.pmed.1003692 (PMC8270422; doi:10.1371/journal.pmed.1003692)
Supplement: S8 Table — (DOCX) [file pmed.1003692.s012.docx]

## Table S8. Validation statistics for CHIME for participants with prediabetes

|  | **Internal validation: CMS** | | **External validation: CHARLS** | |
| --- | --- | --- | --- | --- |
|  | Discrimination:  C-statistic | Calibration: slope/intercept | Discrimination:  C-statistic | Calibration: slope/intercept |
| Mortality | 0.803 (0.784, 0.822) | 0.930/ 0.000 | 0.756 (0.724, 0.797) | 0.910/-0.010 |
| Myocardial infarction | 0.785 (0.745, 0.828) | 1.178/-0.037 | - | - |
| Ischemic heart disease | 0.707 (0.661, 0.747) | 0.574/ 0.017 | 0.722 (0.694, 0.756) | 0.887/ 0.080 |
| Heart failure | 0.826 (0.796, 0.862) | 0.846/-0.012 | - | - |
| Stroke | 0.770 (0.735, 0.805) | 1.160/-0.015 | 0.816 (0.712, 0.945) | -* |
| Peripheral vascular disease | 0.749 (0.655, 0.817) | 0.972/-0.021 | - | - |
| Ulcer of skin | 0.828 (0.770, 0.888) | 0.837/-0.002 | - | - |
| Renal failure | 0.788 (0.752, 0.838) | 0.357/ 0.020 | 0.713 (0.677, 0.744) | 0.933/ 0.055 |
| Cataract | 0.759 (0.733, 0.784) | 1.090/-0.047 | 0.725 (0.672, 0.777) | 0.436/-0.017 |
| Diabetes | 0.567 (0.543, 0.590) | 0.936/ 0.237 | 0.545 (0.514, 0.592) | 0.884/ 0.031 |
|  | **RMSPE** | **R^2^** | **RMSPE** | **R^2^** |
| Overall | 4.58 | 0.798 | 3.89 | 0.832 |

* only three centiles for calibration.
