# Supplementary material for: Development and validation of the CHIME simulation model to assess lifetime health outcomes of prediabetes and type 2 diabetes in Chinese populations: A modeling study
Source: PLoS Med. 2021 Jun 24;18(6):e1003692. doi: 10.1371/journal.pmed.1003692 (PMC8270422; doi:10.1371/journal.pmed.1003692)
Supplement: S9 Table — (DOCX) [file pmed.1003692.s013.docx]

## Table S9. External validation of observed against predicted trial end points across all validation trials by outcome for CHIME, UKPDS-OM2 and RECODe models

|  | **CHIME**  **(Diabetes)** | **UKPDS-OM2** | **RECODe** | **CHIME  (Prediabetes)** |
| --- | --- | --- | --- | --- |
| **Outcome** | **RMSPE (%)** | **RMSPE (%)** | **RMSPE (%)** | **RMSPE (%)** |
| Mortality | 8.60 | 5.76 | 13.40 | 0.68 |
| Heart Failure | 5.46 | - | 5.00 | 0.88 |
| Ischemic heart disease | 2.20 | 5.58 | - | - |
| Myocardial infarction | 8.05 | 4.14 | 6.30 | 0.78 |
| Renal Failure | 3.08 | - | 5.65 | 0.04 |
| Stroke | 1.66 | 1.91 | 2.74 | 1.94 |
| Retinopathy | 2.31 | - | 4.43 | - |
| Ulcer | 0.26 | 0.21 | - | - |
| Amputation | 1.07 | 0.92 | - | - |
| Cataracts | 6.43 | - | - | - |
| Peripheral vascular disease | 0.78 | - | - | - |
| Diabetes | - | - | - | 2.58 |

RMSPE, root mean square percentage error.
