# Supplementary material for: Development and validation of the CHIME simulation model to assess lifetime health outcomes of prediabetes and type 2 diabetes in Chinese populations: A modeling study
Source: PLoS Med. 2021 Jun 24;18(6):e1003692. doi: 10.1371/journal.pmed.1003692 (PMC8270422; doi:10.1371/journal.pmed.1003692)
Supplement: S10 Table — (DOCX) [file pmed.1003692.s014.docx]

## Table S10. External validation of observed against predicted end points by outcome and individual trial for CHIME, UKPDS-OM2 and RECODe models

| **Model** | **Validation study** | | **Outcome** | **Observed (%)** | **Predicted (%)** |
| --- | --- | --- | --- | --- | --- |
| CHIME | ACCORD | control | Mortality | 6.41 | 7.64 |
| CHIME | ACCORD | control | Heart failure | 4.00 | 9.80 |
| CHIME | ACCORD | control | Ischemic heart disease | 11.99 | 7.28 |
| CHIME | ACCORD | control | Myocardial infarction | 6.73 | 2.56 |
| CHIME | ACCORD | control | Renal failure | 2.52 | 4.93 |
| CHIME | ACCORD | control | Cerebrovascular disease | 2.73 | 4.48 |
| CHIME | ACCORD | treatment | Mortality | 6.73 | 7.83 |
| CHIME | ACCORD | treatment | Heart failure | 3.68 | 10.98 |
| CHIME | ACCORD | treatment | Ischemic heart disease | 11.25 | 8.17 |
| CHIME | ACCORD | treatment | Myocardial infarction | 5.89 | 2.79 |
| CHIME | ACCORD | treatment | Renal failure | 2.63 | 4.23 |
| CHIME | ACCORD | treatment | Cerebrovascular disease | 1.58 | 3.87 |
| CHIME | ACE | control | Mortality | 6.91 | 7.85 |
| CHIME | ACE | control | Diabetes | 16.29 | 17.85 |
| CHIME | ACE | control | Heart failure | 2.27 | 3.21 |
| CHIME | ACE | control | Myocardial infarction | 3.40 | 3.08 |
| CHIME | ACE | control | Renal failure | 1.55 | 1.50 |
| CHIME | ACE | control | Cerebrovascular disease | 2.47 | 4.56 |
| CHIME | ACE | treatment | Mortality | 6.80 | 6.93 |
| CHIME | ACE | treatment | Diabetes | 13.71 | 14.05 |
| CHIME | ACE | treatment | Heart failure | 2.06 | 2.86 |
| CHIME | ACE | treatment | Myocardial infarction | 3.81 | 2.77 |
| CHIME | ACE | treatment | Renal failure | 1.34 | 1.32 |
| CHIME | ACE | treatment | Cerebrovascular disease | 2.37 | 4.16 |
| CHIME | ADVANCE | control | Mortality | 8.52 | 8.07 |
| CHIME | ADVANCE | control | Ischemic heart disease | 9.62 | 7.83 |
| CHIME | ADVANCE | control | Retinopathy | 5.11 | 5.61 |
| CHIME | ADVANCE | control | Cerebrovascular disease | 5.41 | 5.32 |
| CHIME | ADVANCE | treatment | Mortality | 7.31 | 7.81 |
| CHIME | ADVANCE | treatment | Ischemic heart disease | 8.41 | 7.33 |
| CHIME | ADVANCE | treatment | Retinopathy | 5.21 | 5.39 |
| CHIME | ADVANCE | treatment | Cerebrovascular disease | 5.11 | 4.78 |
| CHIME | DPP | control | Diabetes | 33.40 | 30.12 |
| CHIME | DPP | lifestyle | Diabetes | 18.30 | 18.84 |
| CHIME | DPP | treatment | Diabetes | 28.30 | 23.88 |
| CHIME | J-EDIT | pooled | Heart failure | 1.43 | 9.18 |
| CHIME | J-EDIT | pooled | Ischemic heart disease | 5.16 | 6.01 |
| CHIME | J-EDIT | pooled | Myocardial infarction | 2.74 | 5.17 |
| CHIME | J-EDIT | pooled | Cerebrovascular disease | 6.48 | 6.80 |
| CHIME | J-EDIT | pooled | Ulcer of the skin | 1.10 | 0.83 |
| CHIME | JDCS | pooled | Mortality | 6.62 | 8.70 |
| CHIME | JDCS | pooled | Ischemic heart disease | 7.86 | 6.49 |
| CHIME | JDCS | pooled | Myocardial infarction | 3.17 | 1.95 |
| CHIME | JDCS | pooled | Cerebrovascular disease | 6.07 | 4.26 |
| CHIME | JPAD | control | Ischemic heart disease | 9.38 | 9.62 |
| CHIME | JPAD | control | Myocardial infarction | 3.91 | 3.34 |
| CHIME | JPAD | control | Cerebrovascular disease | 8.44 | 8.03 |
| CHIME | JPAD | treatment | Ischemic heart disease | 10.00 | 9.67 |
| CHIME | JPAD | treatment | Myocardial infarction | 4.38 | 3.70 |
| CHIME | JPAD | treatment | Cerebrovascular disease | 8.75 | 8.25 |
| CHIME | UKPDS 33 | control | Amputation | 1.67 | 0.38 |
| CHIME | UKPDS 33 | control | Cataracts | 7.29 | 13.94 |
| CHIME | UKPDS 33 | control | Mortality | 19.47 | 11.63 |
| CHIME | UKPDS 33 | control | Heart failure | 3.33 | 2.52 |
| CHIME | UKPDS 33 | control | Myocardial infarction | 16.97 | 1.53 |
| CHIME | UKPDS 33 | control | Renal failure | 0.83 | 4.79 |
| CHIME | UKPDS 33 | control | Retinopathy | 11.66 | 7.61 |
| CHIME | UKPDS 33 | control | Cerebrovascular disease | 5.00 | 4.19 |
| CHIME | UKPDS 33 | treatment | Amputation | 1.05 | 0.25 |
| CHIME | UKPDS 33 | treatment | Cataracts | 5.76 | 11.95 |
| CHIME | UKPDS 33 | treatment | Mortality | 18.74 | 9.41 |
| CHIME | UKPDS 33 | treatment | Heart failure | 3.04 | 4.09 |
| CHIME | UKPDS 33 | treatment | Myocardial infarction | 14.86 | 1.68 |
| CHIME | UKPDS 33 | treatment | Renal failure | 0.63 | 4.37 |
| CHIME | UKPDS 33 | treatment | Retinopathy | 8.69 | 6.54 |
| CHIME | UKPDS 33 | treatment | Cerebrovascular disease | 5.65 | 3.94 |
| CHIME | UKPDS 80 | control | Mortality | 38.64 | 22.88 |
| CHIME | UKPDS 80 | control | Myocardial infarction | 16.03 | 6.04 |
| CHIME | UKPDS 80 | control | Peripheral vascular disease | 3.60 | 2.53 |
| CHIME | UKPDS 80 | control | Cerebrovascular disease | 5.71 | 8.34 |
| CHIME | UKPDS 80 | treatment | Mortality | 35.39 | 22.85 |
| CHIME | UKPDS 80 | treatment | Myocardial infarction | 14.61 | 5.76 |
| CHIME | UKPDS 80 | treatment | Peripheral vascular disease | 2.37 | 2.61 |
| CHIME | UKPDS 80 | treatment | Cerebrovascular disease | 7.60 | 8.64 |
| CHIME | UKPDS 80 | control | Mortality | 35.41 | 22.52 |
| CHIME | UKPDS 80 | control | Myocardial infarction | 14.54 | 5.21 |
| CHIME | UKPDS 80 | control | Cerebrovascular disease | 6.71 | 8.21 |
| CHIME | UKPDS 80 | treatment | Mortality | 31.02 | 21.72 |
| CHIME | UKPDS 80 | treatment | Myocardial infarction | 13.44 | 5.35 |
| CHIME | UKPDS 80 | treatment | Cerebrovascular disease | 5.15 | 8.66 |
| UKPDS | ACCORD | control | Mortality | 6.41 | 10.63 |
| UKPDS | ACCORD | control | Ischemic heart disease | 11.99 | 2.74 |
| UKPDS | ACCORD | control | Myocardial infarction | 6.73 | 5.94 |
| UKPDS | ACCORD | control | Cerebrovascular disease | 2.73 | 2.65 |
| UKPDS | ACCORD | treatment | Mortality | 6.73 | 10.71 |
| UKPDS | ACCORD | treatment | Ischemic heart disease | 11.25 | 2.61 |
| UKPDS | ACCORD | treatment | Myocardial infarction | 5.89 | 6.16 |
| UKPDS | ACCORD | treatment | Cerebrovascular disease | 1.58 | 2.52 |
| UKPDS | ADVANCE | control | Mortality | 8.52 | 11.52 |
| UKPDS | ADVANCE | control | Ischemic heart disease | 9.62 | 3.22 |
| UKPDS | ADVANCE | control | Cerebrovascular disease | 5.41 | 2.98 |
| UKPDS | ADVANCE | treatment | Mortality | 7.31 | 11.17 |
| UKPDS | ADVANCE | treatment | Ischemic heart disease | 8.41 | 3.19 |
| UKPDS | ADVANCE | treatment | Cerebrovascular disease | 5.11 | 2.84 |
| UKPDS | J-EDIT | pooled | Ischemic heart disease | 5.16 | 3.76 |
| UKPDS | J-EDIT | pooled | Myocardial infarction | 2.74 | 8.61 |
| UKPDS | J-EDIT | pooled | Cerebrovascular disease | 6.48 | 7.75 |
| UKPDS | J-EDIT | pooled | Ulcer of the skin | 1.10 | 0.89 |
| UKPDS | JDCS | pooled | Mortality | 6.62 | 11.15 |
| UKPDS | JDCS | pooled | Ischemic heart disease | 7.86 | 4.95 |
| UKPDS | JDCS | pooled | Myocardial infarction | 3.17 | 5.38 |
| UKPDS | JDCS | pooled | Cerebrovascular disease | 6.07 | 2.76 |
| UKPDS | JPAD | control | Ischemic heart disease | 9.38 | 7.13 |
| UKPDS | JPAD | control | Myocardial infarction | 3.91 | 9.39 |
| UKPDS | JPAD | control | Cerebrovascular disease | 8.44 | 5.84 |
| UKPDS | JPAD | treatment | Ischemic heart disease | 10.00 | 7.66 |
| UKPDS | JPAD | treatment | Myocardial infarction | 4.38 | 10.05 |
| UKPDS | JPAD | treatment | Cerebrovascular disease | 8.75 | 6.54 |
| UKPDS | UKPDS 33 | control | Amputation | 1.67 | 0.52 |
| UKPDS | UKPDS 33 | control | Mortality | 19.47 | 12.50 |
| UKPDS | UKPDS 33 | control | Myocardial infarction | 16.97 | 9.16 |
| UKPDS | UKPDS 33 | control | Cerebrovascular disease | 5.00 | 3.13 |
| UKPDS | UKPDS 33 | treatment | Amputation | 1.05 | 0.43 |
| UKPDS | UKPDS 33 | treatment | Mortality | 18.74 | 12.01 |
| UKPDS | UKPDS 33 | treatment | Myocardial infarction | 14.86 | 8.54 |
| UKPDS | UKPDS 33 | treatment | Cerebrovascular disease | 5.65 | 2.92 |
| UKPDS | UKPDS 80 | control | Mortality | 38.64 | 29.05 |
| UKPDS | UKPDS 80 | control | Myocardial infarction | 16.03 | 15.67 |
| UKPDS | UKPDS 80 | control | Cerebrovascular disease | 5.71 | 7.17 |
| UKPDS | UKPDS 80 | treatment | Mortality | 35.39 | 29.04 |
| UKPDS | UKPDS 80 | treatment | Myocardial infarction | 14.61 | 14.88 |
| UKPDS | UKPDS 80 | treatment | Cerebrovascular disease | 7.60 | 7.05 |
| UKPDS | UKPDS 80 | control | Mortality | 35.41 | 28.43 |
| UKPDS | UKPDS 80 | control | Myocardial infarction | 14.54 | 13.30 |
| UKPDS | UKPDS 80 | control | Cerebrovascular disease | 6.71 | 6.43 |
| RECODe | ACCORD | control | Mortality | 6.41 | 10.61 |
| RECODe | ACCORD | control | Heart failure | 4.00 | 6.06 |
| RECODe | ACCORD | control | Myocardial infarction | 6.73 | 10.24 |
| RECODe | ACCORD | control | Renal failure | 2.52 | 5.14 |
| RECODe | ACCORD | control | Cerebrovascular disease | 2.73 | 3.83 |
| RECODe | ACCORD | treatment | Mortality | 6.73 | 10.66 |
| RECODe | ACCORD | treatment | Heart failure | 3.68 | 6.09 |
| RECODe | ACCORD | treatment | Myocardial infarction | 5.89 | 10.24 |
| RECODe | ACCORD | treatment | Renal failure | 2.63 | 5.19 |
| RECODe | ACCORD | treatment | Cerebrovascular disease | 1.58 | 3.93 |
| RECODe | ADVANCE | control | Mortality | 8.52 | 17.39 |
| RECODe | ADVANCE | control | Retinopathy | 5.11 | 10.13 |
| RECODe | ADVANCE | control | Cerebrovascular disease | 5.41 | 5.51 |
| RECODe | ADVANCE | treatment | Mortality | 7.31 | 17.11 |
| RECODe | ADVANCE | treatment | Retinopathy | 5.21 | 10.05 |
| RECODe | ADVANCE | treatment | Cerebrovascular disease | 5.11 | 5.46 |
| RECODe | J-EDIT | pooled | Heart failure | 1.43 | 12.13 |
| RECODe | J-EDIT | pooled | Myocardial infarction | 2.74 | 16.91 |
| RECODe | J-EDIT | pooled | Cerebrovascular disease | 6.48 | 6.27 |
| RECODe | JDCS | pooled | Mortality | 6.62 | 8.49 |
| RECODe | JDCS | pooled | Myocardial infarction | 3.17 | 7.45 |
| RECODe | JDCS | pooled | Cerebrovascular disease | 6.07 | 2.50 |
| RECODe | JPAD | control | Myocardial infarction | 3.91 | 9.07 |
| RECODe | JPAD | control | Cerebrovascular disease | 8.44 | 2.91 |
| RECODe | JPAD | treatment | Myocardial infarction | 4.38 | 9.92 |
| RECODe | JPAD | treatment | Cerebrovascular disease | 8.75 | 3.56 |
| RECODe | UKPDS 33 | control | Mortality | 19.47 | 6.87 |
| RECODe | UKPDS 33 | control | Heart failure | 3.33 | 2.72 |
| RECODe | UKPDS 33 | control | Myocardial infarction | 16.97 | 6.74 |
| RECODe | UKPDS 33 | control | Renal failure | 0.83 | 8.44 |
| RECODe | UKPDS 33 | control | Retinopathy | 11.66 | 6.83 |
| RECODe | UKPDS 33 | control | Cerebrovascular disease | 5.00 | 2.51 |
| RECODe | UKPDS 33 | treatment | Mortality | 18.74 | 7.10 |
| RECODe | UKPDS 33 | treatment | Heart failure | 3.04 | 2.84 |
| RECODe | UKPDS 33 | treatment | Myocardial infarction | 14.86 | 6.97 |
| RECODe | UKPDS 33 | treatment | Renal failure | 0.63 | 8.14 |
| RECODe | UKPDS 33 | treatment | Retinopathy | 8.69 | 6.14 |
| RECODe | UKPDS 33 | treatment | Cerebrovascular disease | 5.65 | 2.64 |
| RECODe | UKPDS 80 | control | Mortality | 38.64 | 17.03 |
| RECODe | UKPDS 80 | control | Myocardial infarction | 16.03 | 15.95 |
| RECODe | UKPDS 80 | control | Cerebrovascular disease | 5.71 | 5.46 |
| RECODe | UKPDS 80 | treatment | Mortality | 35.39 | 15.72 |
| RECODe | UKPDS 80 | treatment | Myocardial infarction | 14.61 | 13.25 |
| RECODe | UKPDS 80 | treatment | Cerebrovascular disease | 7.60 | 4.84 |
| RECODe | UKPDS 80 | control | Mortality | 35.41 | 16.57 |
| RECODe | UKPDS 80 | control | Myocardial infarction | 14.54 | 14.56 |
| RECODe | UKPDS 80 | control | Cerebrovascular disease | 6.71 | 4.73 |
| RECODe | UKPDS 80 | treatment | Mortality | 31.02 | 14.88 |
| RECODe | UKPDS 80 | treatment | Myocardial infarction | 13.44 | 12.73 |
| RECODe | UKPDS 80 | treatment | Cerebrovascular disease | 5.15 | 3.92 |

CHIME, Chinese Hong Kong Integrated Modelling and Evaluation; RECODe, Risk Equations for Complications Of type 2 Diabetes; UKPDS-OM2, UK Prospective Diabetes Study Outcomes Model 2.

ACE, Acarbose Cardiovascular Evaluation; ACCORD, Action to Control Cardiovascular Risk in Diabetes; ADVANCE, Action in Diabetes and Vascular disease: preterAx and diamicroN-MR Controlled Evaluation; DPP, Diabetes Prevention Programme; JDCS, Japan Diabetes Complications Study; J-EDIT, Japan Elderly Diabetes Intervention Trial; JPAD, Japanese Primary Prevention of Atherosclerosis with Aspirin for Diabetes trial; UKPDS, United Kingdom Prospective Diabetes Study.
