# Supplementary material for: Development and validation of the CHIME simulation model to assess lifetime health outcomes of prediabetes and type 2 diabetes in Chinese populations: A modeling study
Source: PLoS Med. 2021 Jun 24;18(6):e1003692. doi: 10.1371/journal.pmed.1003692 (PMC8270422; doi:10.1371/journal.pmed.1003692)
Supplement: Attachment — Submitted filename: Rebuttal letter.docx [file pmed.1003692.s015.docx]

3 March 2021

Dear PLOS Medicine Editors and Reviewers,

Thank you very much for reviewing our manuscript:
“Development and validation of a patient-level model to assess lifetime health outcomes of prediabetes and type 2 diabetes in Chinese populations (CHIME): a modelling study”
(PMEDICINE-D-20-05652R1)

We appreciate that the opportunity to improve our manuscript with the very helpful comments from the editors and reviewers. We carefully revised our manuscript and provided a detailed point-by-point response below.

Sincerely,

On behalf of all co-authors,

Jianchao Quan *-----------------------------------------------------------*

# Requests from the editors:

**Comments from reviewers are generally positive on the study, but reviewers did request quite a bit of clarification on points including: comparing the model to the RECODe model and ENFORCE mode, and UKPDS-OM2l, the fact that all 13 of the outcomes may not have been validated, reporting of associations between risk factors and clinical outcomes being counterintuitive in table S6; whether there should be separate prediction models for prediabetes and T2 diabetes, the issue of missing data at baseline.

*In your revised paper, please clarify if the analytical approach followed here corresponded to one laid out in a  prospective protocol or analysis plan? Please state this (either way) early in the Methods section.

a) If a prospective analysis plan (from your funding proposal, IRB or other ethics committee submission, study protocol, or other planning document written before analyzing the data) was used in designing the study, please include the relevant prospectively written document with your revised manuscript as a Supporting Information file to be published alongside your study, and cite it in the Methods section. A legend for this file should be included at the end of your manuscript.*

*b) If no such document exists, please make sure that the Methods section transparently describes when analyses were planned, and when/why any data-driven changes to analyses took place.*

Response:

We have detailed in the Methods section:

“The analyses per se were not prespecified but have formed part of a multinational research project studying the long-term costs of diabetes care. As part of that work, we needed to undertake risk prediction modelling in order to ultimately assess the net value of medical spending on diabetes care using longitudinal patient-level from multiple health systems in Asia, Europe, and North America [Eggleston, 2020; Eggleston, Newhouse et al. 2009]

More specifically, the analyses for model development were planned after obtaining and reviewing the Hong Kong Hospital Authority CMS data, without which we did not a priori understand or had access to even the data fields and structures available. Initially we planned to validate against simulated cohorts from published trials before we obtained the individual-level CHARLS data. Further analyses on model calibration and comparison with other models were made in response to peer review.”

Eggleston, K., Chen, B. K., Chen, C. H., Chen, Y. I., Feenstra, T., Iizuka, T., ... & Newhouse, J. P. (2020). Are quality-adjusted medical prices declining for chronic disease? Evidence from diabetes care in four health systems. *The European Journal of Health Economics*, 1-14.

Karen Eggleston, Nilay D. Shah, Steven A. Smith, Amy E. Wagie, Kirsten H. Long, Arthur R. Williams, Ernst R. Berndt, Jerome H. Grossman, and Joseph P. Newhouse (the Mayo/Harvard/MIT Collaborative Study Group), 2009. “The Net Value of Health Care for Patients with Type 2 Diabetes, 1997 to 2005,” *Annals of Internal Medicine* 151(6): 386-393.

*-----------------------------------------------------------*

# Comments from the reviewers:

# Reviewer #1:

*"Development and validation of a patient-level model to assess lifetime health outcomes of prediabetes and type 2 diabetes in Chinese populations (CHIME): a modelling study" presents predictive outcome models for diabetic and prediabetic patients, developed from an East Asian (Hong Kong Chinese) population of nearly 100,000 participants. The developed Chinese Hong Kong Integrated Modelling and Evaluation (CHIME) model consists of 13 parametric proportional hazard model-based risk equations, each corresponding to one of thirteen outcomes (all-cause mortality, 4 macrovascular outcomes, 7 microvascular outcomes, development of diabetes). Calibration performance was performed on the development data and outperformed the UKPDS-OM2 significantly. External validation was pursued on the China Health and Retirement Longitudinal Study (CHARLS), and simulated data from 9 published trials (one Chinese, three Japanese, five Western). It was found that in general, CHIME performed better for the East Asian trial data, as compared to the Western data. Online interactive visualization and the risk equation formula were provided.

While the presentation is detailed and fairly comprehensive, there remain some concerns that might be clarified:

1. For the comparison of CHIME vs. UKPDS-OM2 (Table 2), the set of outcomes involved for each model are different (e.g. CHIME includes retinopathy, but not UKPDS-OM2), but a single (aggregated) RMSPE and R^2 are provided for each model. It might be clarified as to whether the RMSPE/R^2 metrics were computed only on the common set of outcomes shared between both models. Ideally, the metrics for each outcome for each model might be provided (as in Table S7)*

Response:

Thank you for this suggestion. We have updated Table S9 to include the metrics for each outcome in each model (CHIME, UKPDS-OM2 and RECODe). We have also updated Table 2 to show the aggregate endpoint metrics (RMSPE/R^2) for the full range of outcomes available (rather than the common set of shared outcomes) for CHIME, UKPDS-OM2 and RECODe models.

*2. For Table S7, trial endpoint metrics might also be provided for individual trials where appropriate, in addition to the aggregate values (for the multiple Japanese/Western trials), especially as it is not clear whether some outcomes might be available in some trials for an aggregate group.*

Response:

In Table 3, we present the endpoint metrics for each individual trial for the CHIME, UKPDS-OM2 and RECODe models, and we also list the available outcomes for each trial.  *3. While UKPDS-OM2 was compared against CHIME for the CMS derivation cohort (where CHIME's superior performance is perhaps expected) and an external CHARLS cohort, a more complete evaluation would also compare CHIME & UKPDS-OM2 on the simulated data from the 9 additional trials. This would better affirm whether CHIME's superior performance is restricted to non-Asian cohorts.*

Response:

Thank you for this suggestion. We have conducted further analyses of the UKPDS-OM2 model against the simulated data from published trials. Since the UKPDS-OM2 model was developed for participants with diabetes, we excluded two trials of prediabetes: ACE and DPP. Among the remaining seven comparable trials of diabetes participants, we found the CHIME model performed better (lower RMSPE and higher R^2) than UKPDS-OM2 in Asian trials generally, but also ACCORD and ADVANCE. The CHIME model performance was lower than UKPDS-OM2 in the UKPDS trials - perhaps to be expected as the UKPDS-OM2 model was developed from the UKPDS data.

*4. In Line 127, it is stated that individuals under the age of 20 at date of onset were excluded. It might be clarified as to whether the onset of diabetes and/or prediabetes is referenced here, and whether the exclusion by age is standard or has some other justification.*

Response:

We have amended the sentence to: “We excluded individuals under the age of 20 at the date of onset of diabetes or prediabetes (whichever is earlier), or with a diagnosis code for type 1 diabetes.”

The exclusion of the diabetes study population by age is frequently used algorithm to classify type 1 and 2 diabetes in electronic health records in the Hong Kong CMS dataset and elsewhere (Ke, 2020; Quan, 2017; Nichols, 2014).

Ke, C. *et al. BMC Med Res Methodol* **20,** 35 (2020). doi: 10.1186/s12874-020-00921-3

Quan, J. *et al. Diabet. Med.* 34, 902-908 (2017). doi: 10.1111/dme.13284

Nichols, G.A. *et al. Am J Epidemiol* 181(1), 32-39 (2014). doi: 10.1093/aje/kwu255

*5. In Line 179, it is stated that the probability (for certain predictors) to be missing does not depend on outcome (for the CMS dataset used for model prediction). Whether this property of missing probability not depending on outcome was empirically confirmed from the CMS data might be explicitly stated, since the relevant citations appear to be to publications that support the use of complete case analysis assuming the stated property.*

Response:

Thank you for raising this point. To clarify this assumption of the stated property, we have added:

Since the CMS dataset accounts for more than 90% of total bed days in the Hong Kong healthcare system during the study period [32], we assumed the health outcome data were essentially complete and therefore missing data in the predictor variables was not dependent on the outcome. Complete case analysis was preferred over multiple imputation as only the predictors have missing values and the probability to be missing does not depend on outcome [32–34]. Individuals in the derivation cohort with missing predictor data at baseline are shown in S3 Table.

[32] Lee PY, *et al. Hong Kong Med J*. 2020;26 Suppl 2: 1–24.

[33] Sterne JAC, *et al*. *BMJ*. 2009;338: b2393–b2393. doi:10.1136/bmj.b2393

[34] Van Buuren S. *CRC press*; 2018.

[35] White IR, Carlin JB. *Stat Med*. 2010;29: 2920–2931. doi:10.1002/sim.3944

*6. The model fitting process in the Statistical methods section (Lines 183-198) might be described in greater detail, possibly in the supplementary appendix. In particular, how did consultation from clinician experts potentially modify the models fitted by backwards selection? And how were models selected by AIC for multiple distributions (exponential, log-logistic, log-normal, Weibull)?*

Response:

We have added to the Methods:
“The model fitting process for the final risk models were based on a combination of backwards selection process using Akaike’s information criterion (AIC) and consultation from clinical experts within the authorship group. Selected variables were assessed for clinical relevance with the outcome and the direction of associations with final selection based on group consensus among the clinical experts.”

We have amended the sentence to: “…models were selected by AIC for exponential, log-logistic, log-normal, and Weibull parametric distributions, where lower AIC was considered to indicate a better model fit.”

*7. For the CHIME model in Figure 1 (Lines 200-222), it is stated that each individual patient is simulated up until death or time horizon. However, from what could be understood, each risk equation gives a probability Prob{T>=t} given patient features, i.e. would return a probability of each outcome in a given year (e.g. 10% chance of mortality for the next year). As such, is the CHIME model stochastic (i.e. simulates a patient's death/outcomes according to a randomly generated value, and thus might produce different outcomes each time it is executed for the same patient), or is some cutoff implemented, e.g. on the threshold of each outcome?*

Response:

Similar to the approach in other diabetes modelling (Tran-Duy A, 2020) to uncertainty and heterogeneity, we addressed first-order uncertainty (stochastic uncertainty) by performing Monte Carlo simulations with sufficient replications for convergence.

Tran-Duy A, *et al. Diabetes Care*. 2020;43: 1741–1749. doi:[10.2337/dc19-2249](https://doi.org/10.2337/dc19-2249)

*8. For Figure 2, only 8 of the 13 outcomes are displayed. Calibration for CHIME vs. Observed risk might be shown for the remaining outcomes, regardless of availability of UKPDS-OM2 data.*

Response:

We have updated (now Figure 3) to display all 13 outcomes.  *9. For the simulation against published trials by modelling (Lines 247-267), the modelling procedure is described as generating each value from its reported mean and standard deviation. However, it appears likely that at least some variables are dependant on each other (e.g. for "duration of diabetes", its distribution might be dependant on/correlated with patient age for example, with older patients tending to have longer durations). It might be clarified as to whether such considerations were considered. If not, it might be considered to incorporate such dependancies by estimating from existing individual data.*

Response:

We were unable to account for these dependencies in the clinical trials as they were not reported, and we lacked access to the individual level data in the trials. However, the duration of diabetes was zero for the UKPDS trials as they were newly diagnosed, and for ACE and DPP trials as the participants had prediabetes.

*10. For the online model at* [*https://jquan.shinyapps.io/CHIME/*](https://jquan.shinyapps.io/CHIME/)*, the predictors appear to have binary values (e.g. Age is either 50 or 60 only). Is this just an interface implementation choice (i.e. the model works when age is say 55 too)?*

Response: This is an interface implementation choice to improve website loading speed. We have updated the website interface to include more choices, such as age 55.  *-----------------------------------------------------------*

# Reviewer #2:

*Comments on "Development and validation of a patient-level model to assess lifetime health outcomes of prediabetes and type 2 diabetes in Chinese populations (CHIME): a modelling study". There are some issues that the authors should take care of:

1. The authors thought that existing predictive outcomes models for type 2 diabetes developed and validated in historical Western populations may not be applicable for East Asian populations, so there is an urgent need to develop a model for predicting progression of diabetes and related outcomes in Chinese and East Asian populations. What is the predictive performance of UKPDS-OM2 for diabetes progression and related health outcomes in previous studies? Does this CHIME model in this study add other predictive variables compared to the UKPDS-OM2?*

Response:

The UKPDS equations has been shown to typically overestimated risk by 2–4 fold in contemporary Western trials and cohorts (van Dieren, 2011; Basu, 2017; Basu, 2018; Pagano 2021) and also in Asian cohorts (Quan, 2019). The UKPDS OM2 were based on historical cohorts (UKPDS began follow-up in 1977), and the misspecification is likely due to changes in diabetes demographics (e.g, younger age at diagnosis) and treatment (e.g, early therapy and aggressive management) in the following decades.

We considered other candidate predictors following a review of other models (Table S1). Compared to UKPDS-OM2, we (like RECODe) added the pharmacotherapy as predicators: insulin, non-insulin hypoglycemic agents, anti-hypertensives and statins.

van Dieren, S, *et al.* *Diabetologia* 2011;54:264-270. doi: 10.1007/s00125-010-1960-0

Basu S, et al. *Lancet Diabetes Endocrinol*. 2017;5(10):788-798. doi: 10.1016/S2213-8587(17)30221-8

Basu S, *et al*. *Diabetes Care*. 2018;41(3):586-595. doi: 10.2337/dc17-2002

Quan J, *et al*. *J Clin Endocrinol Metab*. 2019;104(12):5823-5830. doi: 10.1210/jc.2019-00731

Pagano, E, *et al*. *Diabetes Obes Metab*. 2021;1-8. doi: 10.1111/dom.14311

*2. Figure 1 shows that the individual's updated risk factors and history of events are used to predict the occurrence of outcomes and changes in risk factors in the next annual cycle. Why did this study not consider the changes of predictors and changes in medication use during follow-up (regular medication use or not, etc.) for the construction of the model?*

Response:

We adopted a similar approach to existing diabetes outcomes models such as UKPDS-OM2, COSMO-t1d (Tran-Duy, *Diabetes Care*. 2020) for including time-varying covariates, whereby updated risk factors and history of events are used to predict the occurrence of outcomes and changes in risk factors in the next annual cycle. Other models such as RECODe do not include any time-varying covariates at all. Developing models that considers changes of predictors and changes in medication use during follow-up (regular medication use or not, etc.) for the construction of the model would be an excellent area in further research.

Tran-Duy A, *et al. Diabetes Care*. 2020;43: 1741–1749. doi:[10.2337/dc19-2249](https://doi.org/10.2337/dc19-2249)

*3. The authors included diagnosed with either prediabetes or type 2 diabetes. Why were lifetime health outcome prediction models not developed and validated separately for prediabetes and type 2 diabetes? For the endpoint of diabetes, whether only prediabetes data were analyzed?*

Response:

Since there is a continuum of risk across the spectrum of risk factor values from prediabetes to diabetes, we have integrated the prediabetes and diabetes models as opposed to treating them discretely. We account for the diabetes treatment threshold by including covariates for medication and applying time varying covariates at each yearly cycle at the individual-level. Pharmacological treatment is also sometimes used for prediabetes. An integrated prediabetes and diabetes model is easier for users to apply and ensure consistency in modelling health outcomes and informing health policy.

We only analysed prediabetes data for the endpoint of diabetes. We have presented the validation results of CHIME model separately for diabetes and prediabetes in the CHARLS validation cohort and the simulated cohorts from published trials. Readers are free to solely use the diabetes or prediabetes model results.

*4. Table S3 shows the sample size of CMS database was 1,542,103 while the complete cases database was 97,628. The missing data among CMS participants at baseline, there are many indictors with a higher rate of missing (BMI, HbA1c, blood pressure, etc.). The updated patient characteristics predictors included in this study were used to develop the model rather than the baseline characteristics.*

Response:

Thank you for this comment.

Table 1 shows the baseline characteristics of the 97,628 study participants used to develop the model. We have amended the title of Table 1 to “Baseline characteristics of participants included in the study”; and the title of Table S3 to “Missing data at baseline (CMS dataset)”.

*5. The inclusion and exclusion criteria for cohort studies and RCT are different and RCT has more stringent inclusion and exclusion criteria. The authors used the published baseline characteristics of the trial participants to generate a simulated cohort. For continuous variables, they took the reported mean and standard deviation to randomly generate values; for binary and categorical variables, they took the percentage of participants with the particular status as the sampling probability. Please describe the relevant references for this method.*

Response:

We have included references to diabetes outcomes models using this method of generating simulated cohorts from trials for validation (e.g, CDC-RTI, CORE, BRAVO) in references 8-10:

[8] Hoerger T, et al. RTI Press. 2009. doi: 10.3768/rtipress.2009.mr.0013.0909

[9] McEwan P, et al. Value Health. 2014;17(6):714-724. doi: 10.1016/j.jval.2014.07.007

[10] Shao H, et al. Pharmaco Economics. 2018;36(9):1125-1134. doi: 10.1007/s40273-018-0662-1

Table S1 includes a list of diabetes outcomes prediction models using this method of generating simulated cohorts from trials for external validation and their references: ECHO-T2DM, CDM, MMD CHD, SPHR, MICADO, EBMI and Archimedes.

Using this method of validation (Table 3), the best performing model for UKPDS trial was UKPDS-OM2, and for ACCORD trial was RECODe, which were their respective development cohorts. This supports the face e of our approach.

*6. Figure 2 shows that the CHIME model overpredicted mortality in CHARLS data, the author thought that the lower observed mortality rate attributed to incomplete ascertainment from loss of follow-up as deaths. What is the missing rate of each endpoint indicator during the follow-up of this study?*

Response:

In response to the reviewer’s helpful suggestions, we have updated our calibration plots in Figure 2. We include the missing proportion of each endpoint indicator for CHARLS in Table S4.

*7. Table S6 shows the hazard ratios of predictors on outcome indicators. In this model, hazard ratio of age for each outcome indicators were less than 1 implying that age is a protective factor? Are the results of the indicators included in this study consistent with the results of the previous studies? For eGFR as a categorical indicator, which was used as the reference? This study included demographic indicators as predictors, and the authors should consider the issue of covariance between the individual predictor variables.*

Response:

Thank you and also reviewer 3 (#5) for this comment. We have corrected the title (now Table S7) to “Survival time ratio”, thus values less than 1 indicate shorter survival time. We also have included normal eGFR as the reference case in Table S7. We reviewed the correlation matrices between the predictors and did not find any sign of multicollinearity.

-----------------------------------------------------------

# Reviewer #3:

*The Authors developed and validated a 13-equation simulation model (CHIME, Chinese Hong Kong Integrated Modeling and Evaluation) from a population-based cohort of individuals with prediabetes or type 2 diabetes (97,628 participants maturing 397,617 person-years) which predicts the individual risk of mortality, micro and macrovascular complications and the development of diabetes. External validation of CHIME was pursued in the CHARLS (China Health and Retirement Longitudinal Study; years 2011-2015) and in 100,000 simulated individuals from nine published studies (five of which carried out in Western countries). The data clearly indicate that CHIME is a new validated prediction model for the risk of progression from pre-diabetes to overt diabetes and the risk of diabetes outcomes. The Authors claim that CHIME performs better among Chinese than Japanese and Western populations, thus filling a gap due to the modest applicability among Asians of Western population-derived forecasting models available so far. The authors like to suggest that models such as theirs have the potential to be implemented by policy makers to plan the organization of health services and to possibly invent new strategies.
The paper is well written and sufficiently concise; most of the conclusions are consistent with the results reported. Overall, studies like this are welcome as they provide a well-functioning and validated predictive models that are likely to soon become a "service" to the health care systems and help make a precision medicine-approach a reality.*

Response: Thank you for the positive feedback.

*I have some criticisms referring to the alleged specificity that the authors attribute to their model.
In details:
1. The model used as a reference was the UKPDS-OM2, which is not the best available among those derived from Western populations. In fact, it is a pity the Authors did not consider testing RECODe, a new model reported to perform much better and to work well in samples from both clinical trials and real-life setting (Basu S et al, Lancet Diabetes Endocrinol 2017; 5: 788 and Basu S et al, Diabetes Care 2018; 41:586). The Authors should, therefore, compare CHIME to RECODe. The use of RECODe will also play the important function to investigate if models derived from non-Asian populations cannot be used to predict clinical outcomes in Asian individuals with diabetes, as the Authors suggest both in the Abstract and the Introduction. As far as predicting all-cause mortality is concerned (by far the most important clinical outcome) and along the same line as above, the Authors might be interested to compare their model also to ENFORCE an established, validated and parsimonious model
(Copetti M et al, J Clin Endocrinol Metab 2019, 104: 4900) which has also proven to be easily improved by adding new markers (Scarale MG et al, Diabetes Care 2020, 43:1). Does CHIME outperform ENFORCE in Chinese individuals? As for RECODe, answering this question will improve a lot the relevance of this study.*

Response:

Thank you for this excellent suggestion. We have now included RECODe as a comparison model in addition to UKPDS-OM2. We have updated the manuscript, tables and figures accordingly.

Both the CHIME and UKPDS-OM2 employ agent-based microsimulation modelling to develop parametric models to predict life-time disease progression and incorporate time-dependent biomarkers and disease history. In contrast, the RECODe model uses baseline characteristics without time-varying covariates to predict outcomes at a specified period based on Cox proportional hazard models.

We read with interest the ENFORCE risk equation of 6-years all-cause mortality. The use of novel predictive biomarkers such hs-CRP and serum amyloid P component is a particularly interesting area of research, though sadly we do not have access to these biomarkers in any of our datasets, as they are not yet standard of care for patients with diabetes here. We have included in the discussion:

“New predictive biomarkers such as hs-CRP and serum amyloid P component have improved for mortality prediction for diabetes, and their inclusion may improve predictions for other outcomes [44,45].”

*2. The Authors should report classical measures of calibration and discrimination for each of the 13 risk equations both in the development and in the validation cohorts. RMSPE and R2 which summarized all outcomes cannot be considered exhaustive. E.g. calibration in-the-large, calibration slope are usually used to assess a model's calibration; while survival c-statistic and other are usually used to assess a model's accuracy. RMSPE and R2 measure and overall calibration for all endpoints but, potentially, CHIME could be better than UKPDS-OM2 (or RECODe or ENFORCE) for certain endpoints and worse for other endpoints.*

Response:

We have updated Table 2 to report classical measures of discrimination (c-statistic) and calibration (slope, intercept) for each of the 13 risk equations in CHIME, and the alternative risk equations in UKPDS-OM2 and RECODe.

*3. While 13 outcomes were used in model development, only five (mortality, heart disease, cerebrovascular disease, renal failure and diabetes status) were validated in the second external cohort. If the Author are not able to validate all 13 outcomes, this should be clearly said in the Abstract, results and Conclusions.*

Response:

We have amended the Abstract, Results and Conclusions to clearly state five outcomes were externally validated in the CHARLS cohort and the remaining outcomes were validated against simulated trial cohorts.

*4. In the validation of CHIME against simulated trial cohorts, a wide range of results was obtained. In fact, RMPS ranges 0.48-3.66 and 0.81-8.56 across trials in Japanese and non-Asians patients, respectively. Two of the five trials from the latter subgroup had RMPS well within the range of values observed in Japanese. Do these heterogeneous results allow us to state, as the Authors did (see Abstract and Conclusions), that the use of CHIME is somehow limited to the Chinese and East Asian populations? Any speculation on how to explain such a great variability? Again, addressing this point, like the one above, will increase the overall relevance of the study, placing it in the broader context of the portability of prediction models across diverse genetic, environmental and cultural backgrounds.*

Response:

We have added to the Discussion:

“While in general CHIME performed better in Asian than non-Asian trials, there were two notable exceptions in J-EDIT and ADVANCE. The J-EDIT trial enrolled substantially older participants of 65-84 year olds (mean age 72) compared to the CMS dataset (mean age 61.9). This older age group with higher risk may be closer to UKPDS-OM2 which tends to overestimate risks in trials and cohorts [4,6,7,24]. CHIME also performed well for the ADVANCE trial which may be due to the diverse geographical/ethnic mix of the trial participants with almost 40% of participants from Asia.”

*5. We have serious concerns about Table S6. In detail, the direction of nearly all associations between clinical outcome and established risk factors appears to be the exact opposite of what was expected. For example, the HR of age or duration of disease for mortality or cardiovascular events is <1, thus suggesting that the two risk factors are protective. The same goes for kidney function. How is it possible? We can speculate that these seemingly counterintuitive results depend on the way HRs were calculated, but unfortunately nothing is said in the methods to help address this problem.*

Response:

Thank you and also reviewer 2 (#7) for this comment. We have corrected the labelling (now Table S7) to “Survival time ratio”, thus values less than 1 (e.g. increasing age, duration) does indeed indicate shorter survival time.

*6. Validation of the CHIME model against additional results (eighty) was performed in nine simulated trial cohorts. This cannot be considered an appropriate validation step. Also, if 13 risk equations were developed, how were 80 endpoints (and thus 80 risk equations) validated? In all, this piece of data should be removed.*

Response:

Table S1 includes a list of diabetes outcomes prediction models using this method of validation against simulated cohorts from trials and their references: CDC-RTI, CORE, BRAVO, ECHO-T2DM, CDM, MMD CHD, SPHR, MICADO, EBMI and Archimedes.

In the Methods section, we have included references to diabetes outcomes models using this method of validation (e.g, CDC-RTI, CORE, BRAVO):

Hoerger T, et al. RTI Press. 2009. doi: 10.3768/rtipress.2009.mr.0013.0909

McEwan P, et al. Value Health. 2014;17(6):714-724. doi: 10.1016/j.jval.2014.07.007

Shao H, et al. Pharmaco Economics. 2018;36(9):1125-1134. doi: 10.1007/s40273-018-0662-1

Using this method of validation, the best performing model for UKPDS trial was UKPDS-OM2, and for ACCORD trial was RECODe, which were their respective development cohorts (table 3). This supports the face validity of this approach.

The 80 endpoints refer to the total number of trial endpoints available for validation, rather than the number of risk equations. Each of the 13 risk equations can be validated against cohorts from multiple trials. For example, the mortality endpoint was reported in six trials: JDCS, ACCORD, ADVANCE, UKPDS 33, UKPDS 80, ACE. We believe that reporting this data would be of benefit to readers to enable comparisons as these results are commonly reported in validation studies of other diabetes trials.

*Minor
A. The two samples used for discovery (CMS) and validation (CHARLES) are quite different in terms of several characteristics known to model diabetes risk and its complications, including the rate of prediabetes (51 vs 74%, respectively), current smokers (11 vs 29%), anti-hypertensive treatment (56 vs 22%), statin treatment (14 vs 6%) and, finally, in terms of eGFR (89 vs 103 ml / min). Despite all this, CHIME performs equally well in the two samples, a result that clearly speaks in favor of its portability in different clinical settings. I strongly suggest the Authors to comment on this aspect.*

Response:

Thank you for this comment. Although the average baseline characteristics differ between CMS and CHARLS participants, these characteristics are adjusted for in the predictor variables. CMS data from Hong Kong and CHARLS data from Mainland China are likely to share substantial genetic, environmental and cultural similarities.

*B. The time horizon used to estimate the survival C-statistic has to be reported in the Methods section.*

Response:

We have added the following to the Methods:

“We assessed model discrimination using the C-statistic at 10-years for CMS and 4-years for CHARLS, with confidence intervals estimated from 100 bootstrap replications”

*C. C-statistics should be reported in Table 2 to help comparing different prediction models.*

Response:

We have included c-statistics in Table 2.

_______________________________________

Again, we thank the Editors and Reviewers for the constructive critiques, which has resulted in a clearer presentation and an improved manuscript. We hope that our revision is now acceptable for publication.

Sincerely,

Jianchao Quan
