# Supplementary material for: Development and validation of the CHIME simulation model to assess lifetime health outcomes of prediabetes and type 2 diabetes in Chinese populations: A modeling study
Source: PLoS Med. 2021 Jun 24;18(6):e1003692. doi: 10.1371/journal.pmed.1003692 (PMC8270422; doi:10.1371/journal.pmed.1003692)
Supplement: Attachment — Submitted filename: Rebuttal Letter.docx [file pmed.1003692.s016.docx]

23 April 2021

Dear PLOS Medicine Editors and Reviewers,

Thank you very much for reviewing our manuscript:
“Development and validation of a patient-level model to assess lifetime health outcomes of prediabetes and type 2 diabetes in Chinese populations (CHIME): a modelling study”
(PMEDICINE-D-20-05652R2)

We appreciate that the opportunity to improve our manuscript with the very helpful comments from the editors and reviewers. We carefully revised our manuscript and provided a detailed point-by-point response below.

Sincerely,

On behalf of all co-authors,

Jianchao Quan

-----------------------------------------------------------

# Requests from the editors:

**Comment 1**
 Author summary, Introduction, and throughout the text: Please avoid use of the term “Western” populations- please use European or another term that best fits your intended meaning.

**Response:** We have replaced “Western” with European or North American as appropriate.

**Comment 2**

Methods Lines 114-117: Please be more specific in mentioning these analyses in the published trials (were the 9 trials determined in advance, for example) and the further analyses and comparisons carried out in response to peer review if possible. “Initially we planned to validate against simulated cohorts from published trials before we obtained the individual level CHARLS data. Further analyses on model calibration and comparison with other models were made in response to peer review.”

**Response:** We have revised the methods as follows:

“We planned to validate against simulated cohorts from 9 trials determined in advance from validation studies of existing diabetes outcome simulation models and trials conducted in East Asia (S1 Table). We subsequently obtained individual level data for model validation from the CHARLS cohort. We planned to compare model performance of CHIME with the existing UKPDS OM2 model. Further comparison with the recently developed RECODe model and calibration assessments by slope and intercept were made in response to peer review.”

**Comment 3**

Methods: Line 119: Please fully define the IRB abbreviations here.

**Response:** We have fully defined the IRB abbreviation as follows:

“The analyses used only de-identified data, and the study was approved by the respective institutional review board for each Hospital Authority cluster (Hong Kong East/West, Kowloon Central/East/West, New Territories East/West).”

**Comment 4**
Methods: Line 203-206: Please do not use underline for emphasis.

**Response:** We removed underline from the manuscript.

**Comment 5**
Table 1: Please include a descriptive legend, defining all abbreviations used in the table.

**Response:** We added a descriptive legend to define all abbreviations used in Table 1.

**Comment 6**

TRIPOD Checklist: Thank you for including the TRIPOD checklist. Please replace the page numbers in the checklist with section/paragraph numbers to refer to locations within the text.

**Response:** We have updated the TRIPOD checklist with section and paragraph numbers.

**Comment 7**

Table 3: Please replace the term “Caucasian” with “White” in this table.

**Response:** We have replaced the term “Caucasian” with “White” in Table 3.

**Comment 8**
Table S9: Please define all abbreviations in the legend (IHD, MI, PVD).

**Response:** We defined all abbreviations in the legend for Table S9.

# Comments from the reviewers:

# Reviewer #1:

We thank the authors for addressing the points raised in the previous review round. Some additional clarifications might be considered:

**Comment 1**

Details on the Monte Carlo simulation convergence procedure for patient death simulation might be provided, perhaps in supplementary material.

**Response:** Thank you for this suggestion. We have provided the convergence plots for trial simulations in the supplementary material (S3 Figure). We have also included the following (lines 387-388): “All simulation trial cohorts were checked for convergence of outcomes (S3 Figure).”

**Comment 2**

The individual RMSPEs for each outcome/endpoint corresponding to each individual dataset (as apparently presented in Figure 4, and summarized in aggregate in S9 Table) might be provided, possibly in supplementary material.

Figure 4 might be reworked as there are currently multiple datapoints mapped to multiple outcomes indicated by approximately the same colour (e.g. Amputation/Mortality/Peripheral vascular disease); it might be advised to more clearly specify what each individual datapoint corresponds to (perhaps with differently-shaped datapoints, other markers)

**Response:** Thank you for this helpful suggestion. We have enhanced Figure 4 with differently shaped points as well as multiple colors. We have presented the individual RMSPE for each outcome/endpoint corresponding to each individual dataset in the supplementary materials (S10 Table).

**Comment 3**

In general, the eighty individual endpoint results from the CHIME, UKPDS-OM2 & RECODE validations (as aggregated in Table 3) might be reported, again possibly in supplementary material. While the aggregated RMSPEs provide a general idea of the model accuracy, it would probably be appropriate to know their applicability to individual outcomes.

**Response:** We have presented the individual RMSPE for each outcome/endpoint corresponding to each individual dataset in the supplementary materials (S10 Table).

**Comment 4**

The significant RMSPE differences for CHIME modelling, between the diabetes and prediabetes cohort (S9 Table), might be discussed. For example, for the mortality outcome, diabetes has RMSPE of 8.66 (inferior to UKPDS-OM2), compared to only 0.69 for prediabetes. Does this suggest that the CHIME model is particularly suited to prediabetes cases, and if so, might a separate model specialized towards diabetes cases be warranted?

**Response:** It difficult to draw this conclusion since as you have noted, the aggregate RMSPE depends on the composition of the underlying trials that vary in reported outcomes, length of follow-up, year of enrolment and indeed participants. Details of the trials are provided in Table 3. We have presented plots by each outcome for Asia vs non-Asian trials and, as you have helpfully suggested, the individual RMSPE for each outcome/endpoint corresponding to each individual dataset in S10 Table.

# Reviewer #2:

**Comment 1**

Comments on "Development and validation of a patient-level model to assess lifetime health outcomes of prediabetes and type 2 diabetes in Chinese populations (CHIME): a modelling study".

This study developed an individual-level health outcomes model (CHIME) and compares it with UKPDS-OM2 and RECODe models. CHIME model is a new validated tool for predicting progression of diabetes and its outcomes, particularly among Chinese and East Asian populations. I am satisfied with the author's revisions and recommend the manuscript for publication in the journal. But I still have a simple suggestion:

Table S7 explores the survival time ratios of predictors in CHIME risk equation, please provide a description of the statistical method used here.

**Response:** Thank you for your helpful comments and suggestions. We have added a description to Table S7 legend as follows:

“Survival time ratio (where <1 indicated shorter survival time). All effects are centered on a common scale to allow the relative strength of various predictors to be judged. Survival time ratios vary continuous predictors from the first to the third quartile.^1^”

Harrell FE. Regression Modeling Strategies. 2nd ed. Cham: Springer International Publishing; 2015. (Springer Series in Statistics).

# Reviewer #3:

***Major***

**Comment 1**

The Authors have addressed some of my criticisms but not all of them. In details, calibration and discrimination measures were not reported when prediction models were applied in the simulated cohorts. This is mandatory given that, once again, it is not fair to propose prediction tools without providing their most intrinsic features.

**Response:** Thank you for raising this comment. Individual level data on who develops/does not develop the observed outcomes is required as validation targets against the prediction model outputs to assess discrimination and calibration performance. We present the discrimination and calibration measures for all three prediction models where we had access to individual level outcome data (CMS and CHARLS), and thus individual or group-level validation targets.

We applied the prediction models to simulated cohorts where we were unable to access the individual level trial data. The lack of validation targets by which the model output is compared meant we were unable to match (or group) individuals from the simulated cohort to corresponding “actual” observed outcome rates in the trial participants to measure calibration slope/intercept among subgroups.^1^ As the trials report overall observed risk for each outcome, we were able to present the overall calibration performance measures by outcome for each trial (total of 80 endpoints), namely the root mean square percentage error (RMPSE) shown in Figure 2, Table 3 (by trial), Table S9 (by outcome), and S10 (by individual trial and outcome).

^1^Dahabreh IJ, Chan JA, Earley A, et al. Modeling and Simulation in the Context of Health Technology Assessment: Review of Existing Guidance, Future Research Needs, and Validity Assessment: Agency for Healthcare Research and Quality (US); 2017 Jan. Chapter 4, A Review of Validation and Calibration Methods for Health Care Modeling and Simulation. Available from: https://www.ncbi.nlm.nih.gov/books/NBK424022/

**Comment 2**

Unfortunately, some of the new findings question the quality of the proposed prediction tool. In fact, the calibration intercept is very far from being 0 (i.e. the optimal value) in both development and validation samples for many endpoints. Furthermore, the calibration slope is very far from 1 (i.e. the optimal value) in the validation samples for all endpoints. Overall, this clearly indicates that the prediction tool is not calibrated. Despite this frustrating result, the Authors have not adopted any strategy to correct it, nor have they commented on it fairly. Conversely, in the Discussion Section (lines 415-416) it is stated that "The CHIME model showed good calibration against the CMS development cohort, moderate calibration against CHARLS and better UKPDS OM-2 calibration." Again, this is simply not the case, as the calibration is not good in CMS and very poor in CHARLS. This is a serious flaw and must be discussed openly.

**Response:**

Thank you for raising this point and giving us the opportunity to thoroughly revise our manuscript. We have re-analysed the calibration performance using the methods applied in the RECODe validation studies (Basu et al., 2017a; Basu et al., 2017b), namely assessing the slope and intercept of the line between predicted and observed probabilities of each outcome by deciles of risk. Like the calibration methods applied in the RECODe validation studies, we collapsed decile groups if fewer than 5 events were observed per group to prevent unstable inferences [37]. We also reviewed our analyses and updated results using the most recent CHARLS validation cohort (Wave 4 / 2018 National Follow-up Survey Data released on Sep 23, 2020) that comprised of 6 years of outcome data instead of the previous 4 years (Wave 3 /2015) for longer follow up.

We have amended the Methods as follows:

“We assessed calibration through the slope and intercept of the line between predicted and observed probabilities of each outcome by deciles of risk, with fewer centiles than deciles used if fewer than 5 events were observed per group to prevent unstable inferences [37].”

We have also updated the Results and Discussion accordingly.

Basu S, Sussman JB, Berkowitz SA, Hayward RA, Yudkin JS. Development and validation of Risk Equations for Complications Of type 2 Diabetes (RECODe) using individual participant data from randomised trials. Lancet Diabetes Endocrinol. 2017;5: 788–798. doi:10.1016/S2213-8587(17)30221-8

Basu S, Sussman JB, Berkowitz SA, Hayward RA, Bertoni AG, Correa A, et al. Validation of Risk Equations for Complications of Type 2 Diabetes (RECODe) Using Individual Participant Data From Diverse Longitudinal Cohorts in the U.S. Diabetes Care. 2017; dc172002. doi:10.2337/dc17-2002

[37] Demler OV, Paynter NP, Cook NR. Tests of calibration and goodness-of-fit in the survival setting: O. V. DEMLER, N. P. PAYNTER AND N. R. COOK. Stat Med. 2015;34: 1659–1680. doi:10.1002/sim.6428

***Minor***

**Comment 3**

Surprisingly, the C-statistic for ischemic heart disease went from 0.697 in the development sample to 0.837 in the validation sample. A similar result was also found for the cerebrovascular outcome. This is an unexpected result, albeit a positive one, as the C-statistic is usually higher in the development sample. Authors should address and comment on this result.

**Response:**

The CHIME model was developed on the full CMS cohort from 2006 to 2017; c-statistics for the full sample are presented in Table S5. Since the RECODe model predicts risks at a specified period of 10 years; for comparison, we assessed the CHIME, UKPDS-OM2 and RECODe models against CMS participants enrolled from 2006-2008 and followed until Dec 31, 2017 (Methods/lines 274-275). The results above (Table 3) are for the CMS participants enrolled from 2006-2008. We have added a note to the legend to clarify this in Table 3. We have updated the analyses with the latest CHARLS validation cohort (Wave 4 / 2018). The validation sample is smaller than the development sample, has fewer events with wider confidence intervals and do not show any statistically significant improvements over the development cohort.

**Comment 4**

When using UKPDS-OM2 and RECODe a good calibration is not shown for all results. This problem, which is especially surprising for the RECODe model, should also be adequately addressed and discussed.

**Response:** The poor calibration results for UKPDS-OM2 is similar to previous validation studies comparing RECODe and UKPDS-OM2, that found the UKPDS-OM2 had calibration slopes ranging from 0.06–1.12 in North American trials (Basu et al. 2017). RECODe showed good discriminative performance, often comparable with the CHIME model for many outcomes. RECODe equations were validated previously in predominantly white populations (11,036 white, 2,854 black, and 1,523 Hispanic; Basu et al., 2017a) or populations with few Asian, or indeed East Asian participants (1,746 black in JHS; 369 white, 548 black, 187 Asian, and 451 Hispanic in MESA; Basu et al., 2017b). RECODe differs markedly from the social and historical context of UKPDS cohort (Basu et al., 2017b), both of which differ even more markedly from the Chinese CMS and CHARLS cohorts. Existing literature comparing the epidemiology in Asian to European/North American populations with diabetes have shown certain outcomes are less common, such as myocardial infarction, but other outcomes are more common, such renal failure [3].

We have revised our discussion as follows:

“The UKPDS-OM2 was developed from a 1970s UK cohort and overpredicted mortality and cerebrovascular disease but underpredicted outcomes that are more common in the Asian population such as renal failure; reflecting the differences in epidemiology between East Asian and European/North American populations [3]. The RECODe model developed from North American trial had similar patterns of overpredicting myocardial infarction and cerebrovascular disease but underpredicting renal failure. The overprediction of macrovascular outcome by UKPDS-OM2 could be due to more intensive management in the past decade, such as early initiation and tighter clinical thresholds for hypoglycemic agents, statins, and anti-hypertensives; whereas RECODe was developed from a more recent trial conducted from 2001 to 2009, and consequently better calibration than UKPDS-OM2.

The RECODe model showed good discrimination, often comparable to the CHIME model, but was less well calibrated to the CMS development cohort. Unlike CHIME and UKPDS-OM2, the RECODe model is restricted to predicting risk at a specific time interval of 10-years and does not incorporate time-varying covariates. This limits its applicability for lifetime projections, and flexibility when validating against sample of varying follow-up periods. As expected, the best performing model for cohorts simulated from UKPDS trial was UKPDS-OM2, and for ACCORD trial was RECODe, which were their respective development cohorts, supporting the face validity of this validation approach. The RECODe (ACCORD trial) differs markedly from the social and historical context of UKPDS trial [7]; both of which differ even more markedly from the Chinese CMS and CHARLS cohorts.”

Basu S, Sussman JB, Berkowitz SA, Hayward RA, Yudkin JS. Development and validation of Risk Equations for Complications Of type 2 Diabetes (RECODe) using individual participant data from randomised trials. Lancet Diabetes Endocrinol. 2017;5: 788–798. doi:10.1016/S2213-8587(17)30221-8

Basu S, Sussman JB, Berkowitz SA, Hayward RA, Bertoni AG, Correa A, et al. Validation of Risk Equations for Complications of Type 2 Diabetes (RECODe) Using Individual Participant Data From Diverse Longitudinal Cohorts in the U.S. Diabetes Care. 2017; dc172002. doi:10.2337/dc17-2002

[3] Ma RC, Chan JC. Type 2 diabetes in East Asians: similarities and differences with populations in Europe and the United States. Ann N Y Acad Sci. 2013;1281: 64–91. doi:10.1111/nyas.12098
