# Supplementary material for: Development and validation of the CHIME simulation model to assess lifetime health outcomes of prediabetes and type 2 diabetes in Chinese populations: A modeling study
Source: PLoS Med. 2021 Jun 24;18(6):e1003692. doi: 10.1371/journal.pmed.1003692 (PMC8270422; doi:10.1371/journal.pmed.1003692)
Supplement: Attachment — Submitted filename: Rebuttal Letter.docx [file pmed.1003692.s017.docx]

4 June 2021

Dear PLOS Medicine Editors and Reviewers,

Thank you very much for reviewing our manuscript:
“Development and validation of the CHIME simulation model to assess lifetime health outcomes of prediabetes and type 2 diabetes in Chinese populations: A modelling study”
(PMEDICINE-D-20-05652R3)

We thank the editors and reviewers for their helpful comments. We have carefully revised the manuscript.

Sincerely,

On behalf of all co-authors,

Jianchao Quan

-----------------------------------------------------------

# Requests from the editors:

1. From the Academic Editor: Please address the remaining comment pertaining to the issue of individual-level validation from Reviewer 3 as completely as possible. Validation of models plays an important role for quality assessment of this model. The authors have validated against real-world observational data and also from data reported in other references. As mentioned by reviewer 3, the model the authors propose can potentially be useful for population-level risk prediction of several endpoints considered together. If the issue of validation at the individual level cannot be objectively solved, please discuss this as a limitation and adjust the description throughout the manuscript (e.g. temper the emphasis on individual-level outcomes in the title, abstract, and main text).

**Response:** We have responded to reviewer 3 below and discussed this as a limitation. We have tempered the emphasis on individual-level outcomes in the title, abstract and main text.

2. Title: We suggest revising to: “Development and validation of the CHIME simulation model to assess lifetime health outcomes of prediabetes and type 2 diabetes in Chinese populations: A modelling study”.

**Response:** We have revised the title as suggested.

3. Author Summary: What did the researchers do and find? We suggest replacing “closer” with “stronger” or “more accurate” in the last bullet point, depending on the meaning intended.

**Response:** We have used “more accurate”.

4. Results: Line 382: Please clarify if this should read “...close correspondence with heart failure and ulcer of the skin.”

**Response:** Yes - we have made this clarification.

5. References: Ref 24, 30: Please abbreviate as “Lancet”.

**Response:** We have used this abbreviation.

6. Figure 3: If possible, please choose colors for the plots that do not require distinguishing red/green as this may be difficult for some readers.

**Response:** We have chosen a color-safe palette for the plots.

7. Supporting Information Files: For each of the Figures presented as supporting information files, please incorporate the title and legend into the file along with the figure.

**Response:** We have incorporated the title and legend into the figures in the supporting information.

8. S3 Figure: Please provide a descriptive title for each individual figure within the file.

**Response:** We have provided a descriptive title for each individual figure.

9. S8 Table: Please provide a final version with the comment removed.

**Response:** We have removed the comment.

# Comments from the reviewers:

# Reviewer #1:

The authors have addressed our previous comments.

# Reviewer #3:

I have the same comment as before: the Authors present the finding on collapsed data from trials (e.g. prediction at population level) as a validation of finding at individual data level. I cannot really agree to accept this as a validation of their model. To be clear, the model the Authors propose can potentially be useful for population-level risk prediction of several endpoints considered together. This is not trivial but is simply different from trying to predict individual outcomes in a given patient.

**Response:** Thank you for your helpful comments. We have added to the Discussion as follows:

Lines 413-415: “We validated using both individual-level data in the CHARLS cohort and aggregate-level data using simulated cohorts from nine published trials.”

Lines 462-464: “The CHIME model can be useful for population-level risk prediction of several endpoints considered together rather than at the individual level.”
